# Correction: A survey of biomedical journals to detect editorial bias and nepotistic behavior

**DOI:** 10.1371/journal.pbio.3001525

**Published:** 2022-01-18

**Authors:** Alexandre Scanff, Florian Naudet, Ioana A. Cristea, David Moher, Dorothy V. M. Bishop, Clara Locher

In the Journal selection and description subsection of the Results, there is a calculation error in the sixth and seventh sentences of the second paragraph. The identification of the author with the largest number of research articles is not adjusted for homonymy bias. The correct sentences are:

The author with the largest number of articles in a given journal is a journalist (N = 767) and, after excluding cases of homonymy in two large journals (Physical Review Letters and Scientific Reports), the author with the largest number of research articles is an academic (N = 198). Both authors publish in established journals (The BMJ and J Enzyme Inhib Med Chem, respectively) and are members of their respective editorial boards.
