# Supplementary figures and images for: A survey of biomedical journals to detect editorial bias and nepotistic behavior
Source: PLoS Biol. 2021 Nov 23;19(11):e3001133. doi: 10.1371/journal.pbio.3001133 (PMC8610247; doi:10.1371/journal.pbio.3001133)

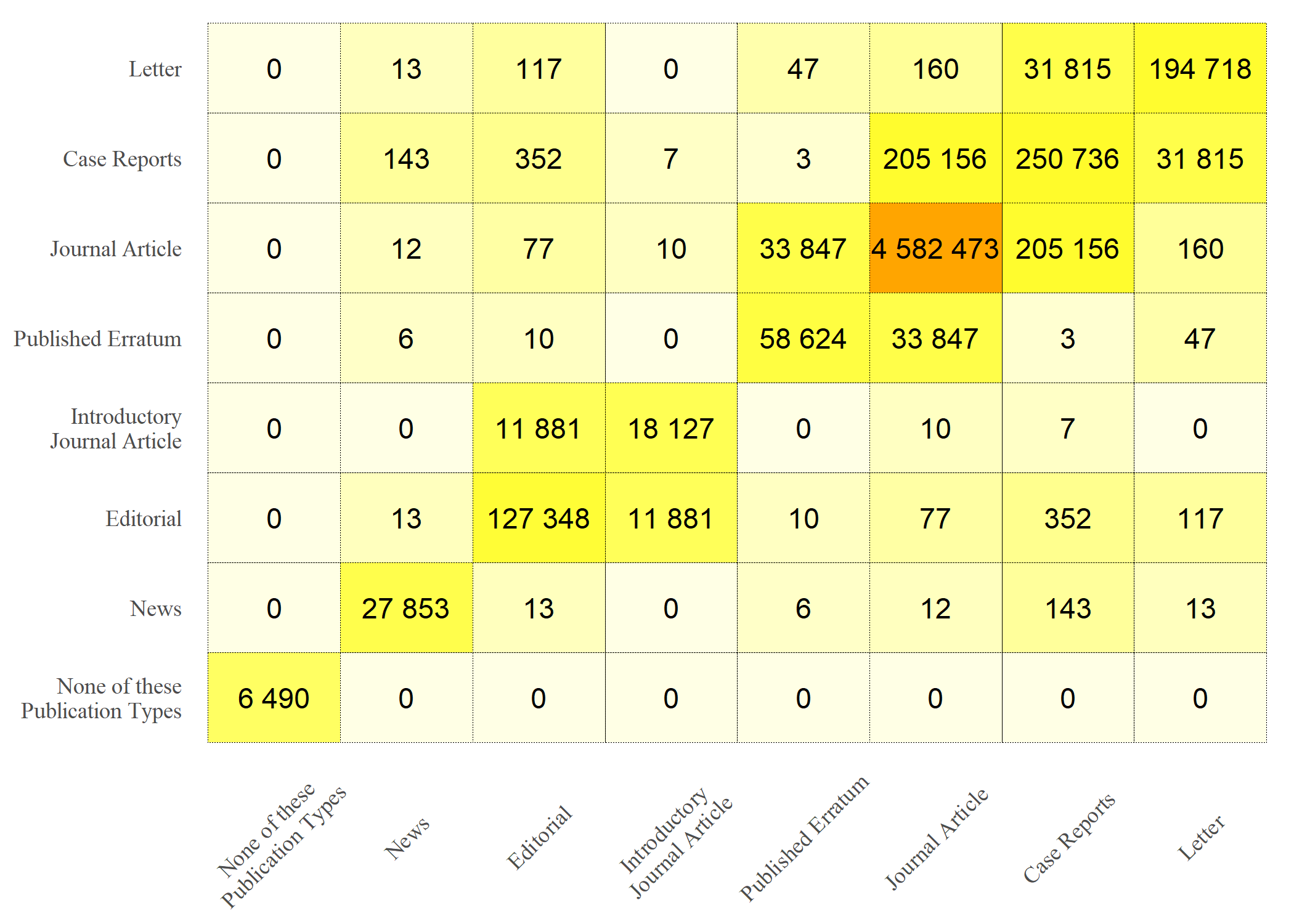

Supplement: S1 Fig — Publication types extracted from MEDLINE Metadata to each article and their co-occurrences, among all journals in the United States NLM catalog having at least one Broad Subject term and having published at least 50 signed articles between 2015 and 2019. The data underlying this figure may be found in https://osf.io/6e3uf/. NLM, National Library of Medicine. (TIF) [file pbio.3001133.s001.tif]

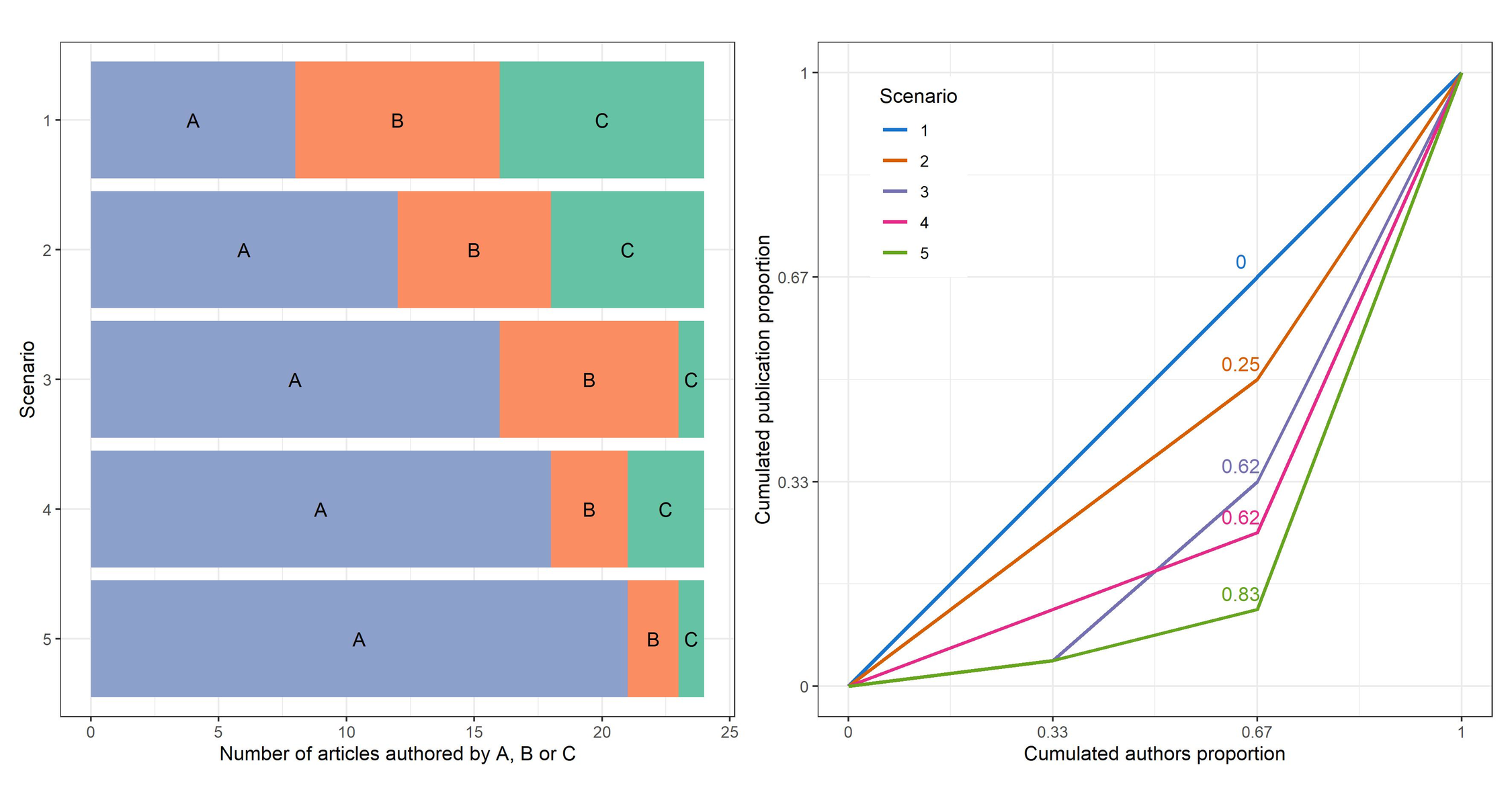

Supplement: S2 Fig — We investigated the level of inequality in the distribution of authorship among authors using the Gini index. This statistical measure is derived from the Lorenz curve and is widely used in econometrics to describe income or wealth inequalities in a given population. In our study, “income” corresponds to the number of articles signed by authors in a given journal, and the “population” is all authors who have published at least one article between 2015 and 2019 in the journal. The Lorenz curve is a graphical representation of the ranked distribution of the cumulative percentage of authors on the abscissa versus the cumulative percentage of authorship distributed along the ordinate axis. In case of complete equality across author (i.e., each author within a journal has published the exact same number of articles), the Lorenz curve would follow the 45 degree diagonal. The further inequality increases (i.e., one author or a group of authors published more articles than others authors), the further the Lorenz curve moves away from this diagonal of equal distribution. The Gini index is a measure of the area between the diagonal of equal distribution and the Lorenz curve, corrected for the number of authors nn−1. In practice, the calculation formula is [2∑iyin∑yi−n+1n]nn−1, where n is the total number of authors, and yi is the number of articles published by author i, with authors sorted in nondecreasing order of article numbers. The Gini index ranges from 0 to 1, with smaller values indicating a more equal distribution of articles across authors and higher values representing greater inequality. As expressed in its formula, the Gini index calculation gives a higher weight to extreme positive values and may be comparable in interpretation to a normalized root-mean-square error against an expected distribution of “all authors appear exactly the same number of times.” As a toy example, hypothetical cases of distribution of authorship between 3 authors totaling 24 authorships [file pbio.3001133.s002.tif]

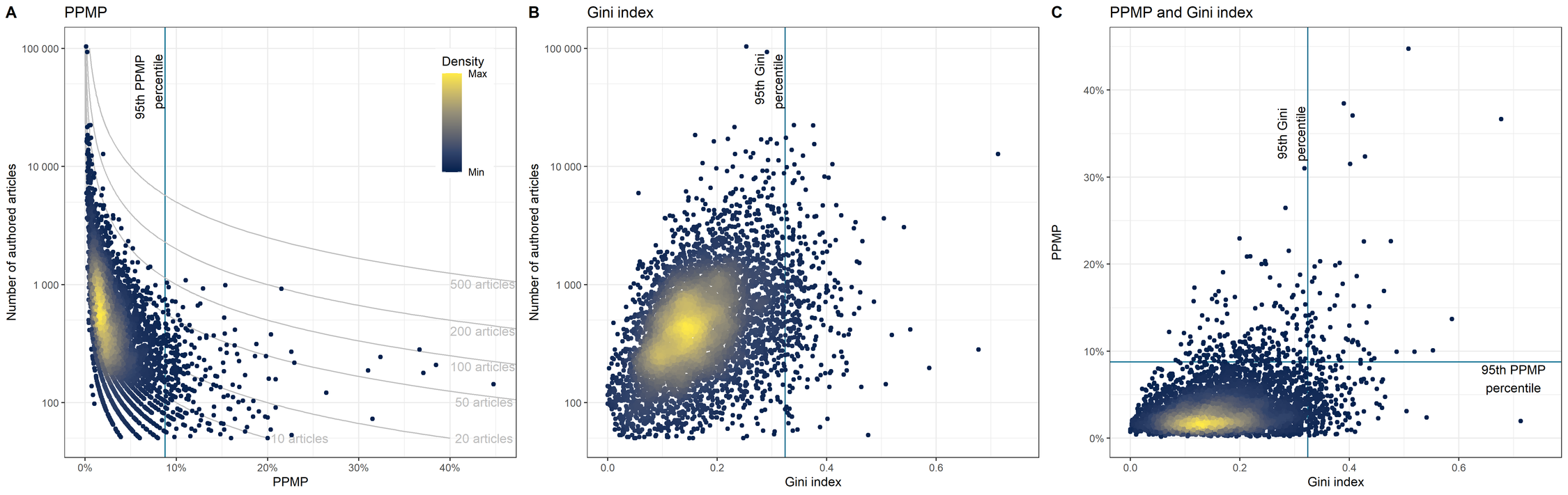

Supplement: S3 Fig — Distribution of the (A) PPMP author(s) and (B) the Gini index in relation to journal size, and (C) comparison between the PPMP and the Gini index, among articles considered as research articles (i.e., original article, case reports, and reviews), published by all journals in the US NLM catalog having at least one Broad Subject term and having published at least 50 signed articles between 2015 and 2019. The data underlying this figure may be found in https://osf.io/6e3uf/. NLM, National Library of Medicine; PPMP, Percentage of Papers by the Most Prolific author. (TIF) [file pbio.3001133.s003.tif]

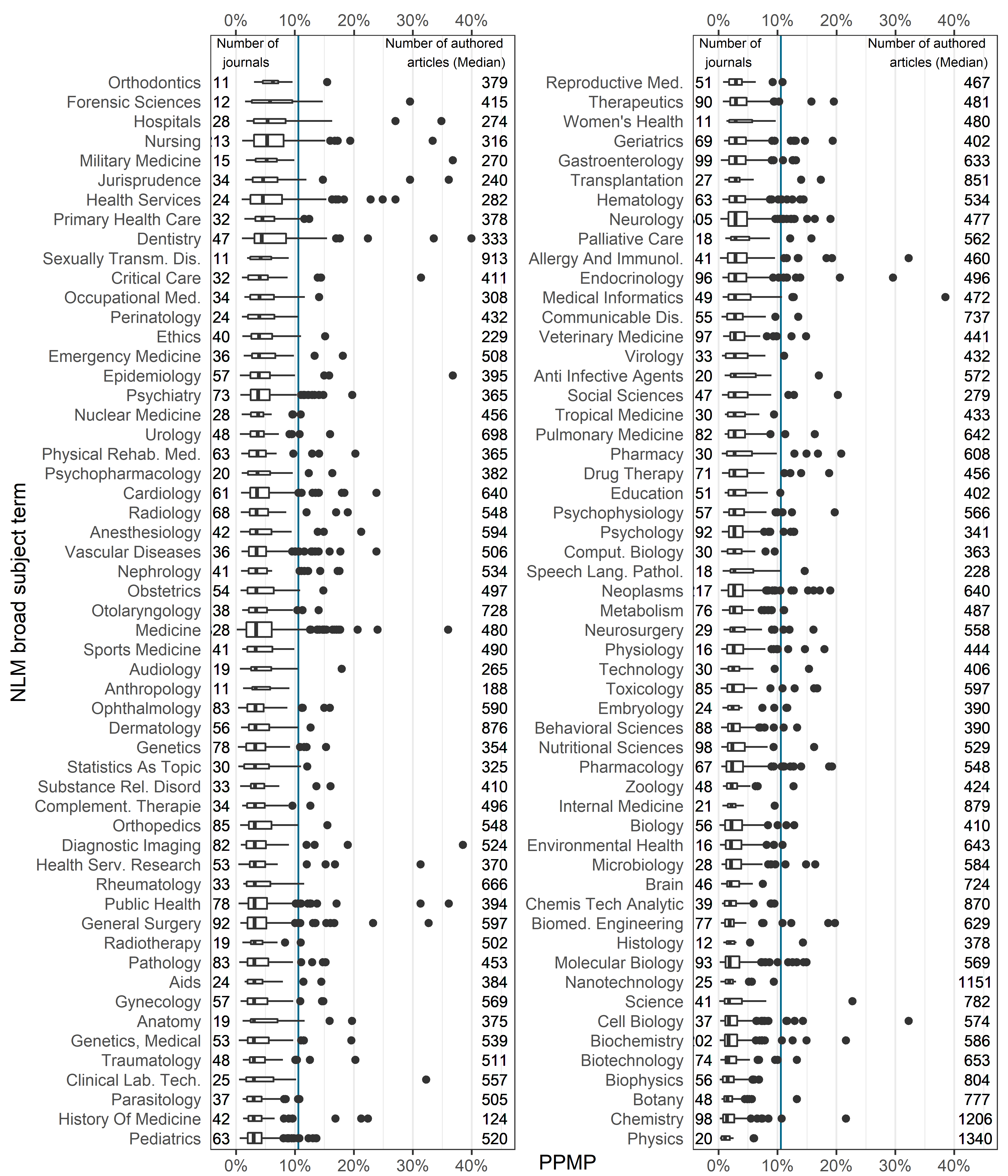

Supplement: S4 Fig — Distribution of the PPMP author for each US NLM broad term represented by at least 10 journals having published at least 50 signed articles between 2015 and 2019. The number of journals covered by a Broad Subject term is shown next to the name of the field of study. Width of the box-and-whisker relative to the number of journals. Vertical line at the 95th percentile of PPMP among journals. The data underlying this figure may be found in https://osf.io/6e3uf/. NLM, National Library of Medicine; PPMP, Percentage of Papers by the Most Prolific author. (TIF) [file pbio.3001133.s004.tif]

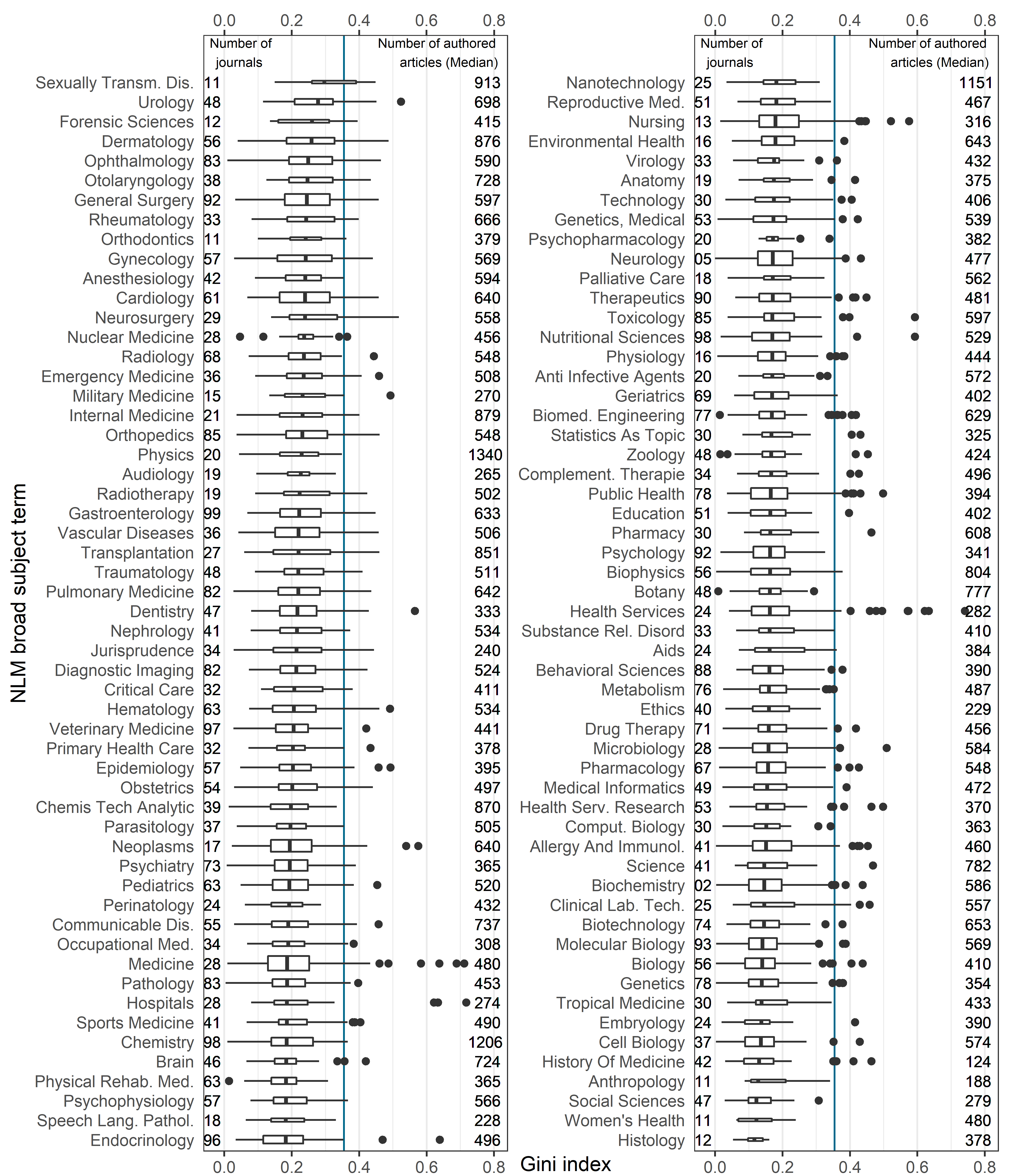

Supplement: S5 Fig — Distribution of the Gini index for each US NLM broad term represented by at least 10 journals having published at least 50 signed articles between 2015 and 2019. The number of journals covered by a Broad Subject term is shown next to the name of the field of study. Width of the box-and-whisker relative to the number of journals. Vertical line at the 95th percentile of PPMP among journals. The data underlying this figure may be found in https://osf.io/6e3uf/. NLM, National Library of Medicine; PPMP, Percentage of Papers by the Most Prolific author. (TIF) [file pbio.3001133.s005.tif]

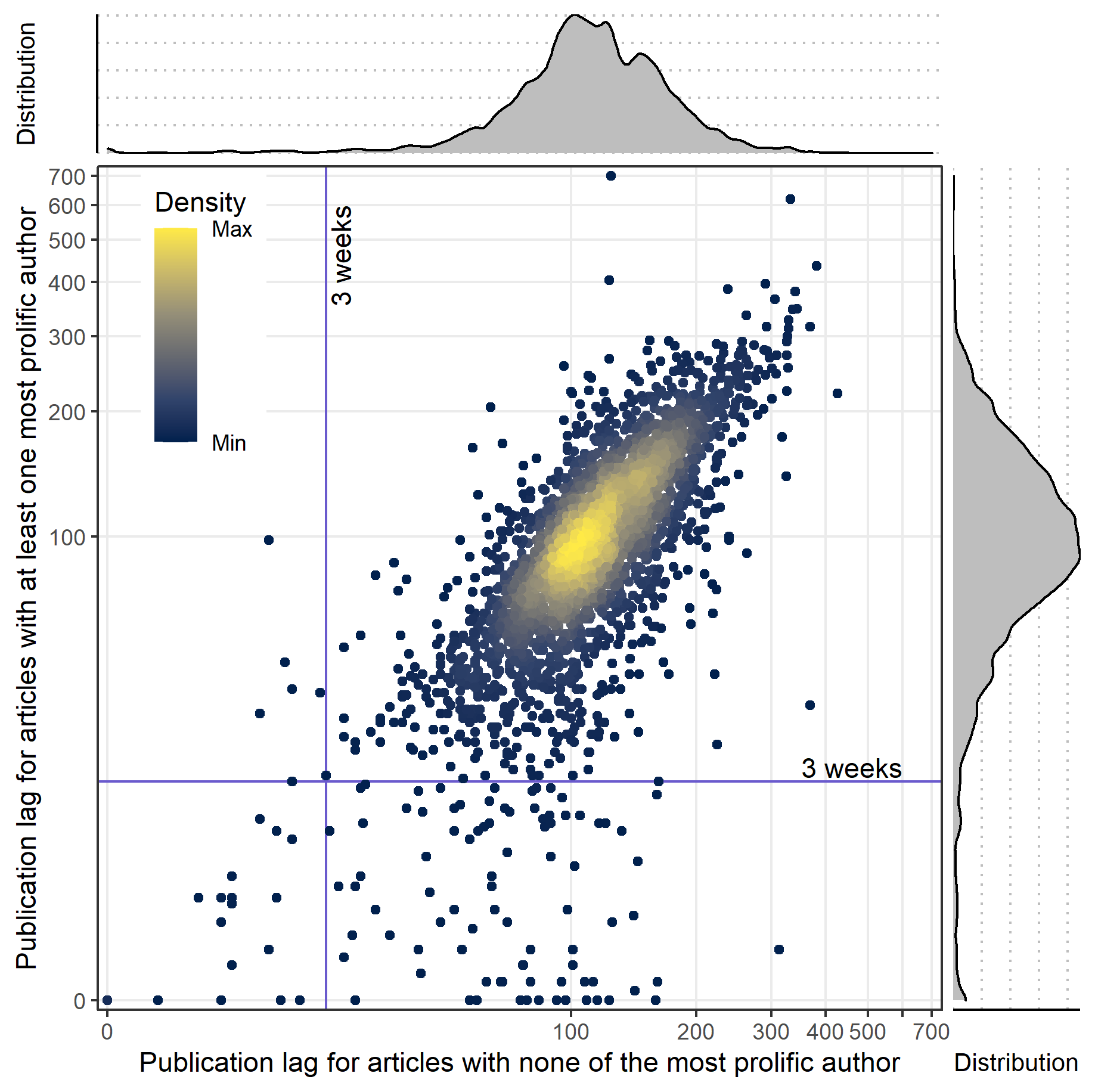

Supplement: S6 Fig — Distribution of the publication lag median (with marginal density plot of distributions) for the subgroup of 2,790 (52.2%) journals reporting submission and publication dates. Publication lag median (in days) are presented for articles signed by the most prolific authors compared to the articles without any of the most prolific authors, among articles considered as research articles (i.e., original article, case reports, and reviews). The data underlying this figure may be found in https://osf.io/6e3uf/. (TIF) [file pbio.3001133.s006.tif]

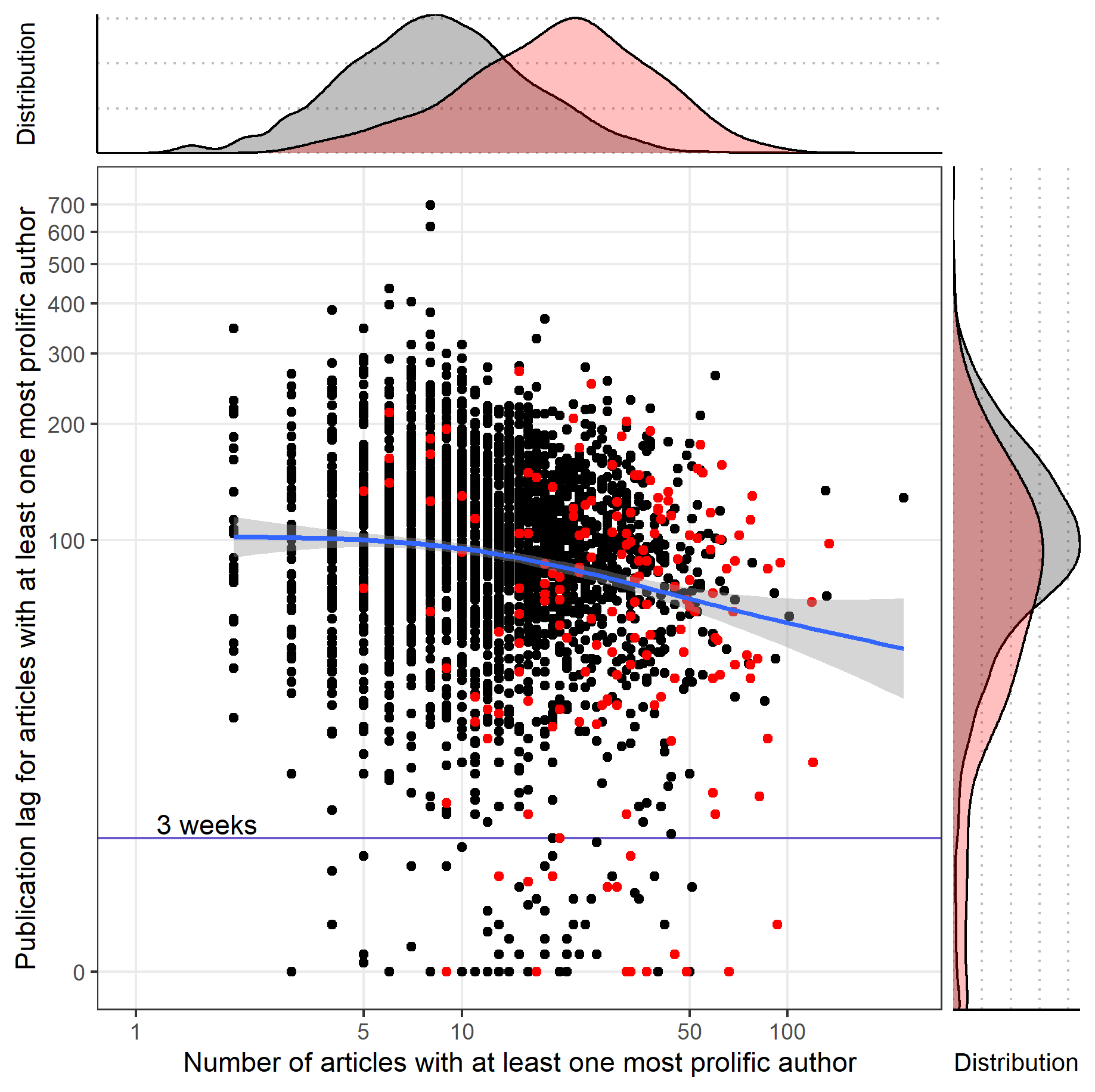

Supplement: S7 Fig — Distribution of publication lag median (with marginal density plot of distributions) and number of articles authored for each of the most prolific authors, across articles considered as research articles (i.e., original article, case reports, and reviews) for the subgroup of 2,790 (52.2%) journals reporting submission and publication dates. The data underlying this figure may be found in https://osf.io/6e3uf/. (TIF) [file pbio.3001133.s007.tif]

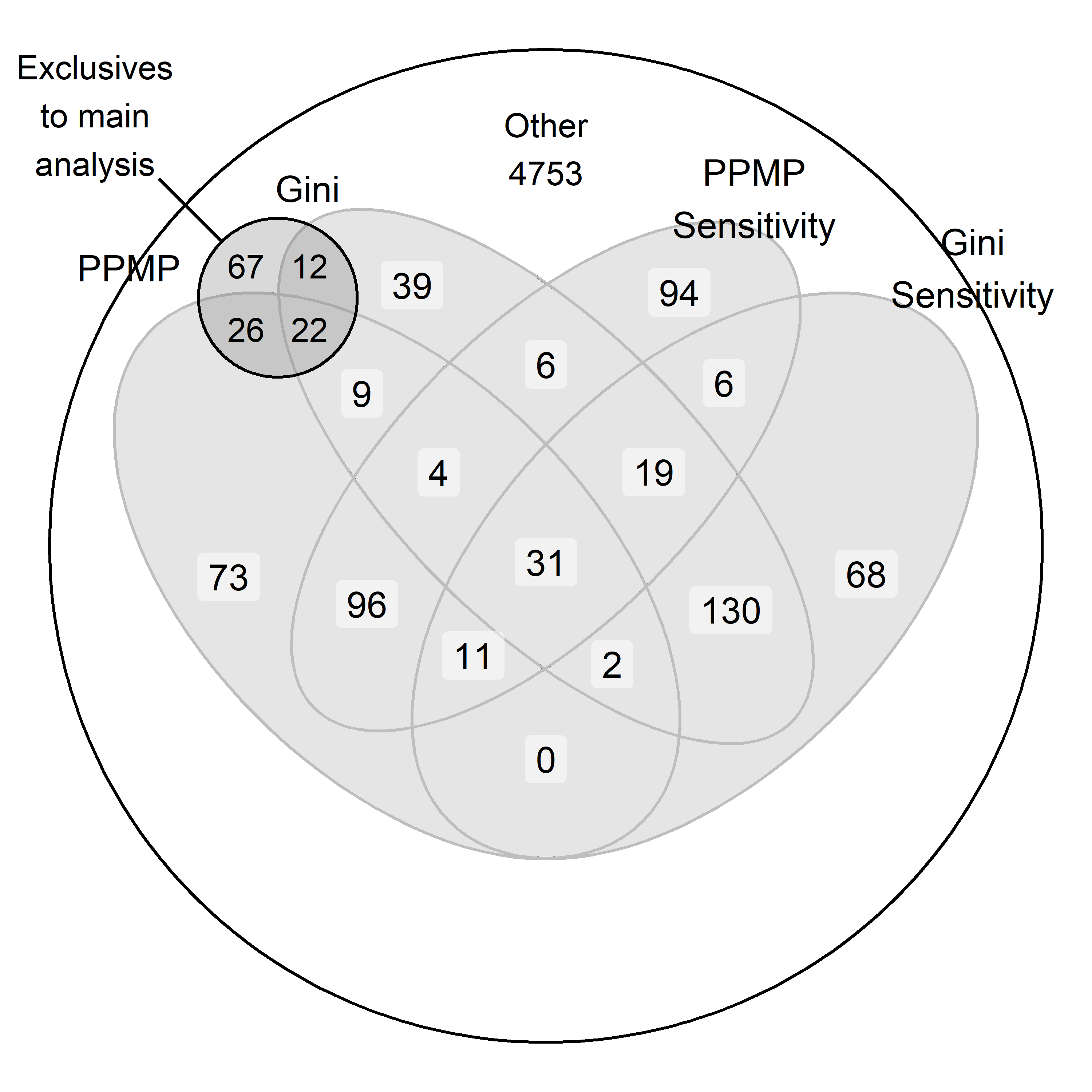

Supplement: S8 Fig — The data underlying this figure may be found in https://osf.io/6e3uf/. PPMP, Percentage of Papers by the Most Prolific author. (TIF) [file pbio.3001133.s008.tif]
