# Supplementary material for: A survey of biomedical journals to detect editorial bias and nepotistic behavior
Source: PLoS Biol. 2021 Nov 23;19(11):e3001133. doi: 10.1371/journal.pbio.3001133 (PMC8610247; doi:10.1371/journal.pbio.3001133)
Supplement: S1 Text — NLM, National Library of Medicine. (DOCX) [file pbio.3001133.s012.docx]

**United States National Library of Medicine catalog journal identification query.**

("acquired immunodeficiency syndrome"[st] OR "aerospace medicine"[st] OR "allergy and immunology"[st] OR "anatomy"[st] OR "anesthesiology"[st] OR "anthropology"[st] OR "anti infective agents"[st] OR "antineoplastic agents"[st] OR "audiology"[st] OR "bacteriology"[st] OR "behavioral sciences"[st] OR "biochemistry"[st] OR "biology"[st] OR "biomedical engineering"[st] OR "biophysics"[st] OR "biotechnology"[st] OR "botany"[st] OR "brain"[st] OR "cardiology"[st] OR "cell biology"[st] OR "chemistry"[st] OR "chemistry techniques, analytical"[st] OR "chemistry, clinical"[st] OR "chiropractic"[st] OR "clinical laboratory techniques"[st] OR "communicable diseases"[st] OR "complementary therapies"[st] OR "computational biology"[st] OR "critical care"[st] OR "dentistry"[st] OR "dermatology"[st] OR "diagnostic imaging"[st] OR "disaster medicine"[st] OR "drug therapy"[st] OR "education"[st] OR "embryology"[st] OR "emergency medicine"[st] OR "endocrinology"[st] OR "environmental health"[st] OR "epidemiology"[st] OR "ethics"[st] OR "family planning services"[st] OR "forensic sciences"[st] OR "gastroenterology"[st] OR "general surgery"[st] OR "genetics"[st] OR "genetics, medical"[st] OR "geriatrics"[st] OR "gynecology"[st] OR "health services"[st] OR "health services research"[st] OR "hematology"[st] OR "histocytochemistry"[st] OR "histology"[st] OR "history of medicine"[st] OR "hospitals"[st] OR "internal medicine"[st] OR "jurisprudence"[st] OR "laboratory animal science"[st] OR "library science"[st] OR "medical informatics"[st] OR "medicine"[st] OR "metabolism"[st] OR "microbiology"[st] OR "military medicine"[st] OR "molecular biology"[st] OR "nanotechnology"[st] OR "neoplasms"[st] OR "nephrology"[st] OR "neurology"[st] OR "neurosurgery"[st] OR "nuclear medicine"[st] OR "nursing"[st] OR "nutritional sciences"[st] OR "obstetrics"[st] OR "occupational medicine"[st] OR "ophthalmology"[st] OR "orthodontics"[st] OR "orthopedics"[st] OR "osteopathic medicine"[st] OR "otolaryngology"[st] OR "palliative care"[st] OR "parasitology"[st] OR "pathology"[st] OR "pediatrics"[st] OR "perinatology"[st] OR "pharmacology"[st] OR "pharmacy"[st] OR "photography"[st] OR "physical and rehabilitation medicine"[st] OR "physics"[st] OR "physiology"[st] OR "podiatry"[st] OR "primary health care"[st] OR "psychiatry"[st] OR "psychology"[st] OR "psychopharmacology"[st] OR "psychophysiology"[st] OR "public health"[st] OR "pulmonary medicine"[st] OR "radiology"[st] OR "radiotherapy"[st] OR "reproductive medicine"[st] OR "rheumatology"[st] OR "science"[st] OR "sexually transmitted diseases"[st] OR "social sciences"[st] OR "speech language pathology"[st] OR "sports medicine"[st] OR "statistics as topic"[st] OR "substance related disorders"[st] OR "technology"[st] OR "teratology"[st] OR "therapeutics"[st] OR "toxicology"[st] OR "transplantation"[st] OR "traumatology"[st] OR "tropical medicine"[st] OR "urology"[st] OR "vascular diseases"[st] OR "veterinary medicine"[st] OR "virology"[st] OR "vital statistics"[st] OR "women's health"[st] OR "zoology"[st])
