# Supplementary material for: A survey of biomedical journals to detect editorial bias and nepotistic behavior
Source: PLoS Biol. 2021 Nov 23;19(11):e3001133. doi: 10.1371/journal.pbio.3001133 (PMC8610247; doi:10.1371/journal.pbio.3001133)
Supplement: Attachment — Submitted filename: renamed_09863.pdf [file pbio.3001133.s013.pdf]

Dear Dr. Roberts,

Thank you very much for the review of our manuscript entitled: ***'Nepotistic journals': a survey of biomedical journals***. We sincerely appreciate all of your and the peer reviewers valuable comments and suggestions. Our point-by-point responses to the reviewers and academic editor comment are described below. Changes in the initial version of the manuscript are highlighted in the revised version.
We look forward to the outcome of your assessment.

Yours sincerely,

Alexandre Scanff, Florian Naudet, Ioana Cristea, David Moher, Dorothy VM Bishop, Clara Locher

---

REVIEWERS' COMMENTS:

Reviewer #1:

The authors perform an analysis related to research integrity, in particular of journals and their editors. For this they use the percentage of papers by the most prolifc author (really a red flag if too high) and the Gini index of contributions over all authors of the journal (a more refined indicator in this case). They further used the median lag time to publication, especially for the most prolific author (another red flag to signal journals with questionable behavior). As a bibliometrician I testify that the authors used the appropriate methods. Of course, the authors could have used another concentration index, but I see no reason why they should in this case.
Thank you for your interest in our manuscript and for these kind comments.

I have two minor observations:

[1] I do not understand the meaning in Fig. 4 of "*number of articles with any most prolific author*". Do they mean "at least one" ?
That is indeed the number of articles with at least one 'most prolific author'. In many journals, there was only one 'most prolific author'. But in some cases, there was more than one author

with the same largest number of published articles. In Fig. 4, publication lag was calculated for all papers with at least one of these most prolific authors. We have edited Figures accordingly: “number of articles with at least one most prolific author”.

[2] the box and whisker graphs pages 26/27 are extremely small. Maybe they can be included, in a larger version, as supplementary material.
The figures are reduced for integration in Word and will be presented as individual 10 000 x 2 000 pixels in the final version.

Reviewer #2:
[identify themselves as Ivan Oransky and Alison Abritis]

[1] Thank you for the opportunity to review this manuscript, which is important as it sounds the alarm on what appears to be very problematic behavior at some journals. We would like to make a number of suggestions for revision.
Thank you for your interest in our manuscript and for your relevant comments and suggestions.

[2] As detailed in our specific comments, we recommend that the authors make it more clear that many of the terms and concepts described in the manuscript have only appeared in their previous non-refereed work, including a preprint. We should stress that we encourage citing such sources, but we also think it is important to be transparent about their status and origin.
We now add references at first mention of new terms. The first paragraph of the Introduction cites Locher et al (previously cited as a preprint, but now peer-reviewed and accepted for publication in the journal *BMJ Evidence Based Medicine* in March 2021 – after submission of this manuscript). The reference has therefore been modified: Locher C, Moher D, Cristea IA, Naudet F. Publication by association: how the COVID-19 pandemic has shown relationships between authors and editorial board members in the field of infectious diseases. BMJ Evid Based Med. 2021 Mar 30.

[3] We would also strongly recommend that the journal invite specialists in economics and statistics to review this paper, particularly the formulas used and subsequent findings. This material is outside of our expertise, but is critical to the paper's conclusions.
Although the Gini index has predominantly been used in economics, where it indicates inequality of income distribution, we do not think it is necessary for an economist to review our paper. We draw parallels between wealth inequality and inequality of access to publication space, and then use the Gini index in a conventional way. It can be computed using DescTools 0.99 package in R: https://CRAN.R-project.org/package=DescTools.
Furthermore, simultaneously with our submission to PLOS Biology, K.L. Hart and R.H. Perlis published in BMJ Open the first bibliometric study which assessed the distribution of authorship using the Gini Index, in a somewhat different context. We now referenced their work in our paper as it provides some sort of external validity to our approach. See below: answers **[4]**, **[7]**, **[11]**.

Introduction:

[4] Paragraphs one and two of the Introduction appear to be a summary of, and borrow language from, the authors' preprint published in July 2020 (reference #1), with little else to support the subsequent hypothesis. It is also unclear from the preprint whether the authors limited their dataset to research articles, or included items such as editorials, which would skew figures for active editors.

The introduction has been modified to make citations from previous works appear more clearly.

Research integrity matters across the research ecosystem. In this process, scientific journal editors are key actors that ensure the trustworthiness of the scientific publication process. But, paraphrasing Dr. Drummond Rennie's famous quote, who is guarding those guardians? [1] Some of our team (CL, IC, DM and FN) had doubts that anyone does such safe guarding in the case of New Microbes and New Infection (NMNI), an Elsevier journal, whose most prolific author, Didier Raoult, co-authored 32% of its 728 published papers [2]. NMNI's editor-in-chief and six additional associate editors of the journal work directly for, and report to, Raoult. Together, these editors authored 44% of the 728 papers published in the journal as of June 25, 2020. We suggested that such 'self-promotion journals' were "*a new type of illegitimate publishing entity, which could have certain key characteristics such as (i) a constantly high proportion of papers published by the same group of authors, (ii) relationships between the editors and these authors, and (iii) publication of low-quality research*" [2]. We applied a preliminary approach to detect these 'self-promotion journals' in the field of infectious disease using a measure easy to compute: the proportion of contributions published in a journal by the most prolific author, i.e. the one who published the most articles in a given time period [2]. In journals publishing more than 50 papers over 5 years, it was rare to see journals where a specific author published more than 10% of the papers, and indeed NMNI was a clear outlier. Note however, this is a crude measure as it is based on all published articles, whatever their type (research, letter, editorial, etc.) and therefore may give high scores for legitimate contributions by active editors.

Coincidently, one of our colleagues (DB) reported a similar analysis for the addiction subfield of psychology in a blog post [3]. This analysis focused on research articles only. She found a bimodal distribution of 'the percentage by the most prolific' measure, identical to that observed for NMNI, with only 3 out of 99 journals having a score over 8%. In two of these journals, the high score was attributable to the same individual, who was on the editorial board of the journal, and who had published together with the editor-in-chief. Bishop also noted that the

same method identified a journal editor, Johnny Matson, who had been found previously to be publishing copiously in journals edited by himself or other editors [3]. Furthermore, for many of these papers, indirect evidence of superficial or absent peer reviews was suspected because of the remarkably rapid turn-around, often within a week or less, between the dates recorded for submission and manuscript acceptance. This circumstantial evidence of unethical editorial practice can only be obtained, however, in journals that report these dates for published manuscripts.

These convergent analyses from different fields suggest that the Percentage of Papers by the Most Prolific author (PPMP) deserves consideration as a potential red flag to identify journals that are suspected of biased editorial decision-making – what we now term 'nepotistic journals'.

We may draw parallels between the PPMP and studies on resource distribution in economics. A highly prolific author who is an outlier on the PPMP measure in effect monopolizes a large proportion of a journal's publications. This analogy supports another, more complex measure, the Gini index [4], used in econometrics to describe resource distribution inequalities. This measure was recently applied in bibliometrics to explore inequality in authorship across authors publishing in high-impact academic medical journals [5]. Applied to our context, it could be used to quantify imbalances in the patterns of authorship within a journal: the values of the Gini index range from 0 (perfect equality in numbers of articles among authors) to 1 (major inequality).

We set out to apply these two indices to a very large dataset of biomedical journals over a 5-year timeframe, to describe outliers using these indices and to describe time intervals between submission and acceptance dates as a potential indicator of unfair or partisan editorial practices.

[1] Rennie D. Guarding the guardians: a conference on editorial peer review. JAMA. 1986;256: 2391–2392.

[5] Hart KL, Perlis RH. Authorship inequality: a bibliometric study of the concentration of authorship among a diminishing number of individuals in high-impact medical journals, 2008-2019. BMJ Open. 2021;11: e046002. doi:10.1136/bmjopen-2020-046002

Regarding the dataset of articles for this preliminary study,

- the primary analysis was computed on **all articles** (including News, Comments, Editorials, or Letters to the editor);
- for the sensitivity analysis, research articles were selected from the field **Publication Type**: articles labelled as '*journal articles*' were included if they were not labelled as '*news*', '*letters*', '*editorial*', or '*comments*'.

In our introduction, we don't discuss the sensitivity analysis but we provide now more details about the analysis set for the main analysis (all articles).
The next part of our answer will provide more details on this as this is a central point. Importantly, both analyses reached the same conclusion. We rather elaborate on this point in the methods and discussion section (see below).

[5] Several phrases were taken directly from the preprint without suitable citation. (e.g. "*We suggest that (1) a constantly high proportion of papers published by a group of authors, (2) particularly in the presence of relationships between the editors and these authors, and (3) publication of low-quality research, are key characteristics of a new type of illegitimate publishing entity, i.e. "self-promotion journals", which deserve further investigation.*") The authors' passage that "*In the field of academic publishing, the term 'self-promotion journal' was coined*" is a good example of one that should be more transparent about the fact that the term was coined by the authors elsewhere and has not yet, best we can tell, been taken up by others.

The introduction has been modified to cite these previous works without ambiguity (the preprint is now published and is carefully referenced). We are now explicit that we coined these terms. In addition, we now refer to the use of Gini by others (actually we identified this reference after we submitted our paper).

Research integrity matters across the research ecosystem. In this process, scientific journal editors are key actors that ensure the trustworthiness of the scientific publication process. But, paraphrasing Dr. Drummond Rennie's famous quote, who is guarding those guardians? [1] Some of our team (CL, IC, DM and FN) had doubts that anyone does such safe guarding in the case of New Microbes and New Infection (NMNI), an Elsevier journal, whose most prolific author, Didier Raoult, co-authored 32% of its 728 published papers [2]. NMNI's editor-in-chief and six additional associate editors of the journal work directly for, and report to, Raoult. Together, these editors authored 44% of the 728 papers published in the journal as of June 25, 2020. We suggested that such 'self-promotion journals' were "*a new type of illegitimate publishing entity, which could have certain key characteristics such as (i) a constantly high proportion of papers published by the same group of authors, (ii) relationships between the editors and these authors, and (iii) publication of low-quality research*" [2]. We applied a preliminary approach to detect these 'self-promotion journals' in the field of infectious disease using a measure easy to compute: the proportion of contributions published in a journal by the most prolific author, i.e. the one who published the most articles in a given time period [2]. In

journals publishing more than 50 papers over 5 years, it was rare to see journals where a specific author published more than 10% of the papers, and indeed NMNI was a clear outlier. Note however, this is a crude measure as it is based on all published articles, whatever their type (research, letter, editorial, etc.) and therefore may give high scores for legitimate contributions by active editors.

[6] The second paragraph of the introduction includes a second reference, to a blog post by a co-author from prior works, and should similarly have more transparent language attached. The authors state, referring to the post's findings: "*Furthermore, many of these papers, with superficial or absent peer reviews, could be detected by the remarkably rapid turn-around, often within a week or less, between the dates recorded for submission and acceptance. This additional evidence of unethical editorial practice can only be obtained, however, in journals that report these dates for published manuscripts.*" We would recommend indicating how these reported dates were interpreted, because for some journals "acceptance date" means the date the paper was accepted *after* having gone through review. The manuscript may have been "accepted" but still required revisions which were done prior to publication. Similarly, a long time period between submission and acceptance does not indicate that a thorough, or even any, peer review was performed. And again, the nature of the publication (research, review, letter, editorial) was not clarified. Were comments related to a single study (e.g., "Authors Reply to Commenter, et al.") written by the same person considered separate articles?

Thank you for this comment. We have clarified these points in the introduction. We have added more detail about the analysis population (research articles) in Dorothy Bishop's blog.

We agree that acceptance might occur before additional revisions have been made but our combined experience in publishing hundreds of articles indicates this outcome is rare (revision after acceptance). Nevertheless, this is surely still an issue, if the interval between submission and acceptance is very brief, and the journal purports to be peer-reviewed. Of course, a long interval does not imply good peer review – in general we suspect there is no correlation between submission-acceptance interval and quality of peer review. The issue becomes of interest only when the interval is so short as to raise questions about whether peer review occurred at all. And we accept that on rare occasions an efficient editor and reviewers may co-occur to enable a remarkably rapid turnaround from submission to acceptance: a single case of this should not be regarded as evidence of malpractice. Furthermore, an editor who did not use any peer reviewers, could very easily game this statistic, by just making the date of acceptance late enough to allay suspicions. And there may also be some journals who

misreport 'submission' dates to refer to date of submission of a revised manuscript, just to make turnaround times at the journal look good. Our focus, however, is on the **distribution** of publication lags: as in the case of Matson, described in DB's blog, we could clearly see that very brief lags between submission and acceptance applied to a specific subset of highly prolific authors (see Figure 2 http://deevybee.blogspot.com/2015/03/will-elsevier-say-sorry.html). We made this point clearer.

Coincidently, one of our colleagues (DB) reported a similar analysis for the addiction subfield of psychology in a blog post [3]. This analysis focused on research articles only. She found a bimodal distribution of 'the percentage by the most prolific' measure, identical to that observed for NMNI, with only 3 out of 99 journals having a score over 8%. In two of these journals, the high score was attributable to the same individual, who was on the editorial board of the journal, and who had published together with the editor-in-chief. Bishop also noted that the same method identified a journal editor, Johnny Matson, who had been found previously to be publishing copiously in journals edited by himself or other editors [3]. Furthermore, for many of these papers, indirect evidence of superficial or absent peer reviews was suspected because of the remarkably rapid turn-around, often within a week or less, between the dates recorded for submission and manuscript acceptance. This circumstantial evidence of unethical editorial practice can only be obtained, however, in journals that report these dates for published manuscripts.

[7] The only other reference in the Introduction, found in the third paragraph, is to a statistical method (Gini) used primarily in economics for studying inequities in resource/income distributions. As noted in our general remarks, we would recommend that the journal seek an reviewer with this kind of expertise.

As discussed above (see reply **[3])**, to our knowledge, K.L. Hart and R.H. Perlis recently published in BMJ Open the first bibliometric study which assessed the distribution of authorship using the Gini Index. This study was published only a few days before our submission, which explains why we missed it and did not include it in the first version of our manuscript. We now refer to their study and the following reference has therefore been added: Hart KL, Perlis RH. Authorship inequality: a bibliometric study of the concentration of authorship among a diminishing number of individuals in high-impact medical journals, 2008–2019. BMJ Open. 2021;11: e046002. doi:10.1136/bmjopen-2020-046002.

These convergent analyses from different fields suggest that the Percentage of Papers by the Most Prolific author (PPMP) deserves consideration as a potential red flag to identify journals that are suspected of biased editorial decision-making – what we now term ‘nepotistic journals’.
We may draw parallels between the PPMP and studies on resource distribution in economics. A highly prolific author who is an outlier on the PPMP measure in effect monopolizes a large proportion of a journal’s publications. This analogy supports another, more complex measure, the Gini index [4], used in econometrics to describe resource distribution inequalities. This measure was recently applied in bibliometrics to explore inequality in authorship across authors publishing in high-impact academic medical journals [5]. Applied to our context, it could be used to quantify imbalances in the patterns of authorship within a journal: the values of the Gini index range from 0 (perfect equality in numbers of articles among authors) to 1 (major inequality).

[5] Hart KL, Perlis RH. Authorship inequality: a bibliometric study of the concentration of authorship among a diminishing number of individuals in high-impact medical journals, 2008-2019. BMJ Open. 2021;11: e046002. doi:10.1136/bmjopen-2020-046002

[8] In the third paragraph, the authors state: "These convergent analyses from different fields *suggest that the Percentage of Papers by the Most Prolific author (PPMP) is a simple measure that can be used as a red flag to identify journals that are suspected of biased editorial decision-making - what we now term 'nepotistic journals.'*" This suggests that there is ample proof that a PPMP is a) "a simple measure" and b) "can be used as a red flag to identify journals that are suspected of biased editorial decision-making." We would recommend that the authors stress in their language that this is not yet a validated measure. (Consider too that journals starting up may have a disproportionately small author list, until well-known enough to attract a variety of new authors.)

Thanks, this clarification was needed to avoid any spin. We agree with you and we modified the introduction accordingly.

These convergent analyses from different fields suggest that the Percentage of Papers by the Most Prolific author (PPMP) deserves consideration as a potential red flag to identify journals that are suspected of biased editorial decision-making – what we now term ‘nepotistic journals’.

We may draw parallels between the PPMP and studies on resource distribution in economics. A highly prolific author who is an outlier on the PPMP measure in effect monopolizes a large proportion of a journal's publications. This analogy supports another, more complex measure, the Gini index [4], used in econometrics to describe resource distribution inequalities. This measure was recently applied in bibliometrics to explore inequality in authorship across authors publishing in high-impact academic medical journals [5]. Applied to our context, it could be used to quantify imbalances in the patterns of authorship within a journal: the values of the Gini index range from 0 (perfect equality in numbers of articles among authors) to 1 (major inequality).

Methods:

Data extraction:

[9] "*by the NLM for journals in the MEDLINE database*" (emphasis ours): Did the authors differentiate between indexed in NLM, PubMed Central, and Medline? The confusion about the differences in terms NLM uses is of course not the fault of the authors, but some clarity would be helpful.

As specified on the following url Journal Subject Terms, "*Not all journals in the NLM Catalog have these broad subject terms in addition to the more specific MeSH terms. Broad Subject Terms are only assigned to MEDLINE journals*".
Therefore, the dataset of journals that we analysed are those **indexed in MEDLINE**. We have added a clarification about this.

"The eligibility criteria for the selection of journals were (i) a biomedical journal referenced in the National Library of Medicine (NLM) in the MEDLINE database, (ii) having at least one 'Broad Subject Term' and, (iii) having published more than 50 papers between January 2015 and December 2019. Broad Subject Terms are Medical Subject Headings (MeSH), terms used to describe a journal's overall scope, and they are defined by the NLM for journals in the MEDLINE database [16]."

[10] What did the authors do about journals having 2 or more Broad Subject Terms (BST). Is there unconsidered duplication within the single BST terms, or was the journal list just considered as a whole without separation per BST?
BSTs were used to retrieve journals indexed in MEDLINE. But for each analysis, each journal appears only once regardless of the number of BST.
The only analysis where a journal might appear more than once is the one for which the distribution of the PPMP and Gini index is analysed for each BST (Web-Appendix 4), in which they will appear once in every one of their BST.
We clarified this point in the method section:

"The eligibility criteria for the selection of journals were (i) a biomedical journal referenced in the National Library of Medicine (NLM) in the MEDLINE database, (ii) having at least one 'Broad Subject Term' and, (iii) having published more than 50 papers between January 2015 and December 2019. Broad Subject Terms are Medical Subject Headings (MeSH), terms used

to describe a journal's overall scope, and they are defined by the NLM for journals in the MEDLINE database [16]. Each journal was analyzed only once regardless of the number of 'Broad Subject Term' associated with the journal (except in subgroup analysis by 'Broad Subject Terms')."

[11] What are considered "*published papers*"? There are plenty of non-research papers indexed - including letters, editorials, and even tables of contents. What means (if any) of exclusion were applied for the bottom limit of 50 publications. This is especially important since the authors needed to revise their protocol to exclude "articles without an author name" - which would be quite an anomaly if concentrating on research and/or review papers.

This is a central point.

For this study, we used data from MEDLINE retrieved *via* easyPubMed, an R interface to the Entrez Programming Utilities. Metadata contain many fields that are described at the following url: MEDLINE/PubMed Data Element (Field) Descriptions.
Among these fields, Publication Type "*describe the form of presentation of materials that are indexed*". The MeSH Pubtypes are listed in the following table:
Publication Characteristics (Publication Types) with Scope Notes

From this information, we faced two difficulties:

1. Metadata from MEDLINE **does not automatically allow the search to focus** in a sensitive and specific manner **on '*research articles*' only**.
2. Metadata from MEDLINE are assigned by NLM indexers (Frequently Asked Questions about Indexing for MEDLINE) who usually follow the journal's practice. As journal practices may differ, the field Publication Type is not standardized from one journal to another.

Partly for these two reasons, we chose to compute the primary analysis on **all articles**, including News, Comments, Editorials, or Letters to the editor. We agree with the reviewers that issues are different depending on the type of articles –research articles or editorials for example– but whatever the type of articles, a high proportion of articles (>10%) raises questions about the plurality of viewpoints and the independence of the journal. At least, these journals deserve a careful inspection.

Once the primary analysis was completed, we performed a sensitivity analysis, focused on papers labeled as '*journal articles*' in PubMed to rule out the possible confounding caused by

other types of article, such as editorials, news and letters. As shown in Web-appendix 1, there is a clear delimitation between journal articles and editorial or news that reassure us about the resolution of the most common situations. Indeed, there are only 12 '*news*', 77 '*editorials*' and 160 '*letters*' that are co-labeled with '*journal articles*'. This is less true in the case of '*comments*': among the 455 147 '*comments*', 83 609 (18.4%) were also labeled '*journal articles*'. However, comments that were also labeled journal articles were not excluded from this sensitivity analysis, as they may represent a very wide range of works.

Still focusing on the 'journal article' category may lead to some false positives. We are well aware of this as our preprint was covered in a Science story that picked a journal with a high PPMP (Journal of the American Dental Association (JADA)). Computation of the metrics was correct. But the author Romina Brignardello-Petersen authored 475 of 1475 articles, or roughly 33%, of articles published in JADA between 2015 and 2019. However, these papers were not submitted as original research articles subject to peer review. 'Rather, Brignardello-Petersen was the section editor responsible for producing the "Clinical Scans" feature that appeared in each issue of JADA during that period. Clinical Scans summarized articles in the professional literature other than JADA to keep JADA readers abreast of important advances in oral-health research.' This is a central point that is also pointed by reviewer 2.

We also pointed this out in our manuscript "*among these 108 authors, this procedure also enabled identification of the 6 most prolific authors, who were professional journalists for whom high productivity is of course not an indicator of any academic misconduct, as they are professionals paid by the journal and not academics. The two proposed indicators, and their current calculation, should therefore not be used indiscriminately but could rather serve as a screening tool for potentially problematic journals that may then require careful exploration of their editorial practices.*" But in our opinion, this point was not clear enough.

We have therefore edited the text in depth (see the discussion section) and we have also clarified our methods. We have revised our sensitivity analysis to limit the number of false positives. We have also extracted information for outliers' journals identified in the sensitivity analysis and that we have analysed the overlap between both analyses.
Please also note that this change to our protocol was:
- Agreed by the team and registered on the OSF (https://osf.io/6e3uf/) prior any new analysis;
- Tracked in our manuscript as a change to the initial protocol;
Please also note that the results remains largely unchanged as they resulted in small numerical changes.

Therefore, in order to refine the sensitivity analysis and to make it more focused on articles eligible for peer review, the selection of articles was revised by adapting methodology from Hart et al. described in two bibliometric studies [5,17]. Compared to their method, the two differences are that we choose to (i) keep reviews (systematic reviews are original articles and review articles contain material that needs to be peer reviewed) and case reports and (ii) exclude articles without abstracts. The objective of this selection is to retain: original research article, case reports and reviews and to exclude other contributions such as comments, letters or editorials.
The text was edited accordingly in both.

A/ The method section

For the primary analysis, these two outcomes (PPMP and Gini) were computed for all papers (including research articles, editorials, comments, etc.), and for a sensitivity analysis they were computed only for research articles (using the NCBI publication type). In line with previous works [5,17], articles were considered as research articles were included if (i) the **Publication Type** field was coded '*Journal Article'*, (ii) the **Abstract** field was not empty. Furthermore, articles were not considered as research articles if **Publication Type** field was coded with the following label: '*Comment*', '*Letter*', '*Editorial*', '*Published Erratum*', '*News*', '*Introductory Journal Article*', '*Biography*', '*Portrait*', '*Congress*', '*Interview*', '*Retraction of Publication*', '*Personal Narrative*', '*Retracted Publication*'', '*Patient Education Handout*', '*Lecture*', '*Autobiography*', '*Clinical Conference*', '*Classical Article*', '*Address*', '*Legal Case*', '*Expression of Concern*', '*Festschrift*', '*Overall*', '*Bibliography*', '*Corrected and Republished Article*', '*Interactive Tutorial*', '*Duplicate Publication*', '*Directory'*, '*Newspaper Article'*, '*Periodical Index'*, '*Dictionary*'.

[5] Hart KL, Perlis RH. Authorship inequality: a bibliometric study of the concentration of authorship among a diminishing number of individuals in high-impact medical journals, 2008–2019. BMJ Open. 2021;11: e046002. doi:10.1136/bmjopen-2020-046002
[17] Hart KL, Perlis RH. Trends in Proportion of Women as Authors of Medical Journal Articles, 2008-2018. JAMA Intern Med. 2019. doi:10.1001/jamainternmed.2019.0907

B/ The change to the initial protocol section.

"Some practical unforeseen challenges arose in our research because a few articles unexpectedly lacked author names (i.e. articles without authors), which precluded them from contributing to the numerator of PPMP or to the Gini index. We therefore amended our definitions to make it explicit that the PPMP denominator was defined as the number of articles

with at least one identified author rather than all published articles, and journals were included only if they had published 50 articles with author names rather than all published articles.
The 3-week threshold used to describe publication lag as being suggestive of unduly rapid or absent peer review was not initially specified in our protocol, and was arbitrarily added for descriptive purposes. Three weeks seems plausible for a thorough review process while expedited reviews in a few days can be suspicious if there is a pattern of fast reviews – and especially if those fast reviews applied selectively to certain favoured authors). We also explored the relationship between publication lag for articles authored by any of the most prolific author (s) and the number of papers authored by these authors. The description of the outlier journals with respect to the membership of any of the most prolific author(s) on the editorial board was added a posteriori for exploratory purposes.
We initially planned to focus our sensitivity analysis on 'journal articles' only. During the peer review process, following a Science's new [23], it appeared clear that this category was not specific enough. The protocol was therefore edited (https://osf.io/6evmz/) with a better definition of 'research articles'. We have also described the overlap between both analysis and added more emphasis on the sensitivity analysis by describing a random sample of outliers in this analysis (it was not part of our initial protocol)."

[23] O'Grady C. Journals singled out for favoritism. In: Science | AAAS [Internet]. 25 Feb 2021 [cited 13 Jul 2021]. Available: https://www.sciencemag.org/news/2021/02/journals-singled-out-favoritism

[12] How did the authors find all the articles per author? Was it through a NLM search via Pubmed or through the individual journal's separate table of contents? Not all journals list all articles within Pubmed - how was the comprehensiveness of the article check ensured?

Authors' names were extracted alongside other articles' metadata and the number of articles of a journal presenting its name was computed for each author. It means that articles were identified in the NLM search and not in each journal's table of content.
Discrepancies in author name between multiple journals were of no impact for our analysis and discrepancies in a given journal were considered low (clerical errors or a potential change in name reporting rules) and too costly in terms of false positive to attempt to implement a correction. However, they were still a possibility for homonyms. As stated in the text, we have tried to clarify this in the more qualitative analysis of outlier journals.

We clarified this point in the limitation section:

"Importantly, our automated calculations rely on the articles identified through a NLM search and it is possible that not all journals list all articles within Pubmed. Although this is likely rare, we cannot exclude that certain authors use somewhat different name (e.g. change of name after marriage). We could not explore this bias that would have resulted in an underestimation of our indicators. More importantly, these calculations carry a risk of inaccuracy as a result of homonymy. Misidentification and/or merging of author names could bias the PPMP and the Gini index in both directions, and the risk of merging increases when only the initial or first-name is known, and in the case of authors with similar names. The greater risk of homonyms could partially explain the increased Gini index values for larger journals, without reference to a tendency to editorial misconduct. Our analysis of the random sample of outliers enabled a disambiguation procedure consisting in inspecting qualitatively the most prolific authors. Only 1 out of 108 'most prolific' authors within a given journal was considered to be at being at high risk of homonymy. Among these 108 authors, this procedure also enabled identification of the 6 most prolific authors, who were professional journalists for whom high productivity is of course not an indicator of any academic misconduct, as they are professionals paid by the journal and not academics. The two proposed indicators, and their current calculation, should therefore not be used indiscriminately but could rather serve as a screening tool for potentially problematic journals that may then require careful exploration of their editorial practices."

[13] "*Publications reprinted in several journals (e.g., PRISMA statements published in 6 different journals to promote dissemination) did not receive special treatment, and no correction was applied, as each article was only examined in relation to its publication journal.*" How many of these cases occurred? Did the authors examine whether excluding these reprints affected any of the findings? It does not seem safe to assume that it would have no effect.

We don't know how many of these cases occurred (likely rarely) but our analysis concerns authorship *within* a single journal, the fact that one article was published in several journals does not modify our analysis. We have not edited the manuscript regarding this point.

Index Calculation:

[14] What did the authors do to confirm that similar/same names were the same person, or that different names were really different people (i.e., referring primarily last name/first name swaps - those using somewhat different names are likely rare, although they do exist).

It is possible that some authors used somewhat different names. This may result in false negative results for these authors (i.e. if those are PPMPs those would not be flagged and the Gini would be lower). We have no option to explore this. We have added some words in the limitation section.

"Importantly, our automated calculations rely on the articles identified through a NLM search and it is possible that not all journals list all articles within Pubmed. Although this is likely rare, we cannot exclude that certain authors use somewhat different name (e.g. change of name after marriage). We could not explore this bias that would have resulted in an underestimation of our indicators. More importantly, these calculations carry a risk of inaccuracy as a result of homonymy. Misidentification and/or merging of author names could bias the PPMP and the Gini index in both directions, and the risk of merging increases when only the initial or first-name is known, and in the case of authors with similar names. The greater risk of homonyms could partially explain the increased Gini index values for larger journals, without reference to a tendency to editorial misconduct. Our analysis of the random sample of outliers enabled a disambiguation procedure consisting in inspecting qualitatively the most prolific authors. Only 1 out of 108 'most prolific' authors within a given journal was considered to be at being at high risk of homonymy. Among these 108 authors, this procedure also enabled identification of the 6 most prolific authors, who were professional journalists for whom high productivity is of course not an indicator of any academic misconduct, as they are professionals paid by the journal and not academics. The two proposed indicators, and their current calculation, should therefore not be used indiscriminately but could rather serve as a screening tool for potentially problematic journals that may then require careful exploration of their editorial practices."

In the other hand, it is possible that homonyms may be merged in the same person. We believe that the risk of this occurrence is higher. As we already state in the manuscript, we explored this for the MPAs explorer in our qualitative analysis. Specifically, when using the WoS Author Search tool there can be only one author found, in which case the problem was trivial, or multiple authors records found publishing in journals in the same field, from one or few similar affiliations, and compatible full name (if only initials were present in the extracted data) or middle name (if full name was present). We agree with reviewer 2 that this should be more explicit in the method section (as this was just presented in the results section).
We have added some details in the method section.

"Outliers were defined as journals with a PPMP value and/or the Gini index above their respective 95th percentiles in the principal analysis (i.e. on all articles). For pragmatic reasons

a sample of 100 outlier journals was randomly selected (first sorted by full name, in alphabetic order, and randomly sampled using a random number generator with a seed arbitrarily set at 42; R function sample_n in dplyr package).

One reviewer (AS or FN or CL) manually extracted characteristics related to the journal impact factor (Web of Science: WoS), open access policies (WoS and Directory of Open Access Journals), open peer review policies (Publons - Clarivate), the most prolific authors' h-index (WoS) and presence and role (i.e. editor in chief or board member) on the editorial board of the journal (journal or publisher website). Where this information was available, we made a distinction between advisory boards (that were not considered in the analysis) and editorial boards. For the year 2019, the metrics ‘self-citation boost’ (i.e. number of self-citing articles over number of non-self-citing articles) and ‘skewness & non-article inflation’ (i.e. impact factor minus median of citations for an article, over median of citations for an article) was computed according to Ioannidis and Thombs [6]. Importantly, this extraction allowed for qualitatively exploring the possibility of homonyms between authors by comparing names, affiliations, research field, and all available qualitative information on NLM and WoS (using WoS Author search tool). When a doubt persisted, we used Google to identify the author at risk of homonymy and their credentials.”

[15] "*When there was more than one author with the same largest number of published articles, they were all considered as the "most prolific" authors.*" If "most" is to be applied to more than one, perhaps a better term applies.

We have maintained the term 'most prolific', as we think it is clear from our description that this rank could be shared by more than one person if they had the same number of papers in the journal.

[16] Use of the Gini index should be assessed by a reviewer familiar with its use and application.

Please refer to answer **[3].**

[17] "*For the primary analysis, these two outcomes (PPMP and Gini) were computed for all papers, and for a sensitivity analysis they were computed only for papers labelled as 'journal articles' (using the NCBI publication type).*" Please explain the difference between "all papers" and for "journal articles."

Refer to answer **[11]**

For the primary analysis, these two outcomes (PPMP and Gini) were computed for all papers (including research articles, editorials, comments, etc.), and for a sensitivity analysis they were computed only for research articles (using the NCBI publication type). In line with previous works [5,17], articles were considered as research articles were included if (i) the **Publication Type** field was coded '*Journal Article'*, (ii) the **Abstract** field was not empty. Furthermore, articles were not considered as research articles if **Publication Type** field was coded with the following label: '*Comment*', '*Letter*', '*Editorial*', '*Published Erratum*', '*News*', '*Introductory Journal Article*', '*Biography*', '*Portrait*', '*Congress*', '*Interview*', '*Retraction of Publication*', '*Personal Narrative*', '*Retracted Publication*", '*Patient Education Handout*', '*Lecture*', '*Autobiography*', '*Clinical Conference*', '*Classical Article*', '*Address*', '*Legal Case*', '*Expression of Concern*', '*Festschrift*', '*Overall*', '*Bibliography*', '*Corrected and Republished Article*', '*Interactive Tutorial*', '*Duplicate Publication*', '*Directory'*, '*Newspaper Article'*, '*Periodical Index'*, '*Dictionary*'.

We hope that this is now clearer. As you will see, we provided more details in the discussion.

Description of outlier journals

[18] The term "*outlier*" could be misleading, as clearly this is referring to a specific group of journals created by specific measured behaviors rated along a particular scale, and "*outliers*" typically refer to statistical anomalies not generally expected to be part of a group.

We thank the reviewers for this comment. With respect, we do not agree that the term outliner is unique to statistics. As such, we have maintained its use.

[19] We will defer to the statisticians in evaluating the true randomness of the sample. However, first alphabetizing journal names (with or without "The" in the title?), then selecting a seed set of 42 suggests "pseudo-randomization," not true randomization. Consider also that journals may avoid names/titles starting with letters towards the end of the alphabet and it becomes even less truly random.

We respectfully disagree. We were required to set a seed for reproducibility purposes. Journals names were used without any modification and ordered to allow for reproduction of the random sampling.

Rows of the table containing outlier journal names were then selected regardless of the character string values but only regarding the row number, using R standard random generator Mersenne-Twister, widely used and approved for less demanding application than cryptography. Allowing reproduction required setting a random seed and 42 had been chosen at the protocol phase as non-singular because : it is popular the choice between 0 and 1 pair may be discussed; there is only one 42. Alexandre Scanff performed this randomization. He always uses this seed when he needs to set a seed, regardless of the data. It may sound unusual to the reviewer, but using the same seed everytime assure that the result is not chosen for the study.

Regarding the number 42 itself, as described in the link above, it is a meme older than the world wide web itself and comes from Douglas Adams 1978's radio comedy and 1979's novel "*The Hitchhiker's Guide to the Galaxy"*, in which it is "*The Answer to the Ultimate Question of Life, The Universe, and Everything*", given by the supercomputer Deep Thought after millions of year of computation. When tell that The Answer makes no sense, Deep Thought simply retorted that we did not understand the Question. The wittiness of the exange and its centrality in the English-packed humor story has engraved the number 42 in many minds, then as now. Please note that it was deemed by the academic editor that the method was correct ("*the seed number chosen is as obvious as right.)*". As such, we have not edited the text regarding this point.

Data analysis

[20] See notes elsewhere about obtaining a review from someone with appropriate expertise in statistics or economics.
Refer to answer **[3]**

Protocol Changes

[21] "*because some articles unexpectedly lacked author names,*" again requires clarification as to the types of publications included as "*article*."

In fact, when we performed our automatic extraction we identified articles with no author name. It was rare. In general, these singular articles were authored by the journal editorial board, but there was no author. We therefore had to excluded these articles. We have added some clarifications.

"Some practical unforeseen challenges arose in our research because a few articles unexpectedly lacked author names (i.e. articles without authors), which precluded them from contributing to the numerator of PPMP or to the Gini index. We therefore amended our definitions to make it explicit that the PPMP denominator was defined as the number of articles with at least one identified author rather than all published articles, and journals were included only if they had published 50 articles with author names rather than all published articles."

[22] "*The 3-week threshold used to describe publication lag as being suggestive of unduly rapid or absent peer review was not initially specified in our protocol, and was added for descriptive purposes.*" What is the basis of using 3 weeks as an arbitrary delineator? Is it just an assumption, or is there literature to show a relationship between 3 weeks and the quality of peer review? If so, include a reference. If not, say so and provide a justification for the time limit.

We agree with the reviewer that this cut-off was arbitrary. As authors ourselves, we are not agnostic to the interval between manuscript submission and acceptance. We typically wait months between submission and a decision from a journal. We think in general, one can never be certain that a rapid review is problematic. This is one of those things where one should look for a pattern. Less than a week between submission date and acceptance data is highly suspicious and highly unusual, because in that interval the editor would have had to look at the paper, identify and contact reviewers, and get reviews back. Two weeks would also be remarkably quick. Three weeks starts to look more plausible for a thorough review process, but we think it would be unusual if more than one reviewer was involved to get turnaround that fast, especially when one takes into consideration the fact that it is often difficult to find reviewers to review a paper, so several people have to be asked. As noted above, an unusually fast review time could have an innocent explanation (e.g., review of a COVID-19 vaccine trial), but one would start to be suspicious if there were a pattern of fast reviews – and especially if those fast reviews applied selectively to certain favored authors.
Our preferred approach, as written in our paper, is to make it explicit that this cut-off was presented only for descriptive purposes. We now insist on the fact that it is arbitrary and we provided some more information to contextualize this.

The 3-week threshold used to describe publication lag as being suggestive of unduly rapid or absent peer review was not initially specified in our protocol, and was arbitrarily added for

descriptive purposes. Three weeks seems plausible for a thorough review process while expedited reviews in a few days can be suspicious if there is a pattern of fast reviews – and especially if those fast reviews applied selectively to certain favoured authors). We also explored the relationship between publication lag for articles authored by any of the most prolific author(s) and the number of papers authored by these authors. The description of the outlier journals with respect to the membership of any of the most prolific author(s) on the editorial board was added a posteriori for exploratory purposes.

Results:

Journal Selection and Description

[23] With such a broad range of numbers of articles published, might there not be a masking effect in journals with higher rates of publishing - causing lower-publishing-rate journals to show a greater number of PPMPs? Would it not have been more effective to break the journals into groups based on rates of publishing?

This is a very interesting comment. In fact, we addressed it in our manuscript.
Having a careful look at Figure 2 (panel A / PPMP), one may see that we present this information with lines that correspond to the number of publications by the most prolific author.

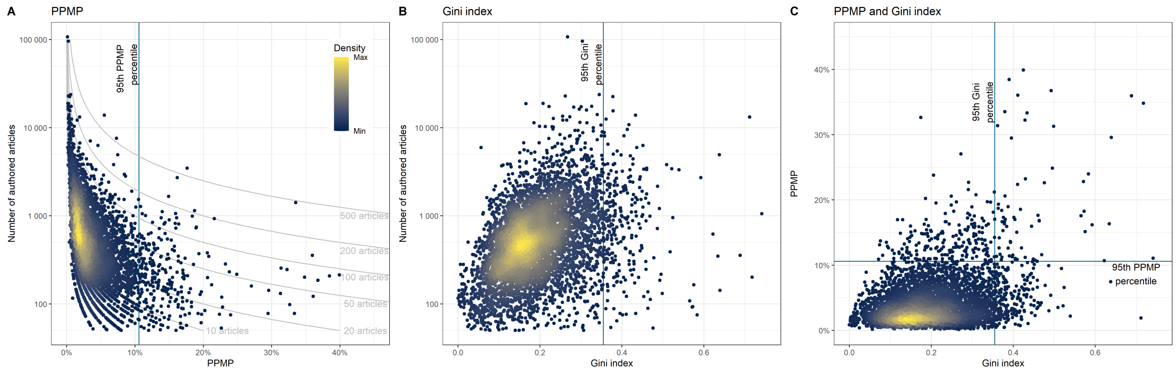

We have made this more explicit in the text, in line with a comment by the editor.

"For the principal analyses based on all articles published during the 2015–2019 period, the PPMP median was 2.88% (IQR 1.71% to 4.91%) and the 95th percentile of the PPMP value was 10.6%. Figure 2A details the PPMP and numbers of published outputs for all journals. It also shows that the number of publications a prolific author in a given journal can result in different PPMP depending on the number of papers published in this journal. The most prolific author(s) in each journal published a median of 14 articles (IQR 8 to 25). For 1022 journals (19%), there was more than one author with the same largest number of published articles. Over the 2015–2019 period, the Gini index median was 0.183 (IQR 0.131 to 0.246), and the 95th percentile was 0.355. Figure 2B details the Gini indices and numbers of published outputs for all journals. Figure 2C presents the correlation between the PPMP and Gini indices. This correlation was 0.35 (95CI 0.33 to 0.37).

For both indices, there were no meaningful differences between index values across years, with the 95th percentile ranging from 10.1% to 11.4% for the PPMP and 0.212 to 0.224 for the Gini index (Web-Appendix 2)."

In addition, Gini does not focus on a single author and may limit this problem.

We are reluctant to add a new analysis that was not pre-specified in our protocol at this time. Rather, we have added some words about this point in the discussion section as it will surely be an important point to consider if one wants to develop further these indices.

"The PPMP and the Gini index explore complementary patterns of asymmetry in publishing patterns. The PPMP reflects author practices while the Gini is more sensitive to groups of highly prolific authors. The Gini index has one advantage over the PPMP, in that it is less constrained by the total number of articles published. If a journal publishes a very large number of papers (i.e. 1000 articles over 12 months), it becomes increasingly implausible that a single prolific author could account for 10% or more of them, as there is a natural upper limit to how many papers any one individual can author. Conversely, for a journal that publishes very few papers, an author could be identified as above the 95th centile with a relatively modest number of publications. In other words, there might be a masking effect in journals with higher rates of publishing causing lower publishing-rate journals to show a greater number of PPMPs. Further developments may consider breaking the journals into groups based on rates of publishing and/or using crude number of publications by the most prolific author."

Description of the Indices

[24] Statistics- defer to a specialist for review.
Refer to answer **[3]**

[25] Slightly more than half the sample did not provide submission/acceptance rates. This suggests it is problematic to draw conclusions about "*publication lag*" calculations and comparisons
It is problematic insofar as we are forced to work with a reduced sample size, and we may therefore miss some journals with suspiciously short publication lags. However, it does not detract from the fact that, where we can study publication lags, we show this measure could be useful. Indeed, our analysis highlights the variability in editorial/publisher practice in how

the timescale of submission and publication is reported, and justifies the suggestion that publishers should adopt more uniform practices in this regard.

We have added extra-caution in our limitation section about the analysis of publication lag. We emphasize now more that this relies on a subgroup and that this subgroup may surely differ from the whole population of journals in terms of editorial standards. In a sense, transparency concerning the reporting of publication dates can be associated with somewhat better editorial standards. We have one reference about this point [7].

[7] Sarigöl E, Garcia D, Scholtes I, Schweitzer F. Quantifying the effect of editor–author relations on manuscript handling times. Scientometrics. 2017;113: 609–631. doi:10.1007/s11192-017-2309-y

We have edited the text accordingly to avoid any spin in our discussion:

"For the subgroup of 2 725 (49.8%) journals reporting submission and publication dates, the time-lag for the most prolific author(s) was shorter, suggesting that for certain journals, that are outliers on PPMP and/or Gini, peer reviews may have been absent or only superficial for prolific authors. However, for the most prolific authors, the publication lag decreases with the number of articles published across all journals and not solely in the sub-sample of outliers. This suggests that our description of these outliers based on PPMP could be only the tip of the iceberg, capturing solely the most extreme cases of hyper-prolific publication in a given journal. Because not all journals report publication lag data, this finding is necessarily based on a subgroup. This could introduce bias if journals reporting publication dates differ from those that do not in terms of factors such as size or monitoring of the publication process [7]."

Description of outliers and identification of nepotistic journals

[26] "*206 based on the PPMP and the Gini index considered separately, and 68 based on both indices.*" If the PPMP and the Gini index each identified 206 different journals, and only agreed on 68, can the authors comment on the significant difference?

The congruence of PPMP and the Gini index will be affected by the fact that these indices are somewhat oppositely associated to the journal size. The median of the number of authored articles over 5 years 170 for the outliers on the PPMP only compared to 1061 for outliers on the Gini index only. It is 241 for the outliers on both PPMP and Gini. As exposed in the

discussion, both measures may need to be considered to give a better flaovur of a given journal practices.

As explained before, we have added much more detail in the new version about overlap of analysis and we have proposed a new figure. This figure also details:
- Overlap of indices (PPMP VS Gini) ;
- Overlap of analyses (all articles VS research articles only).

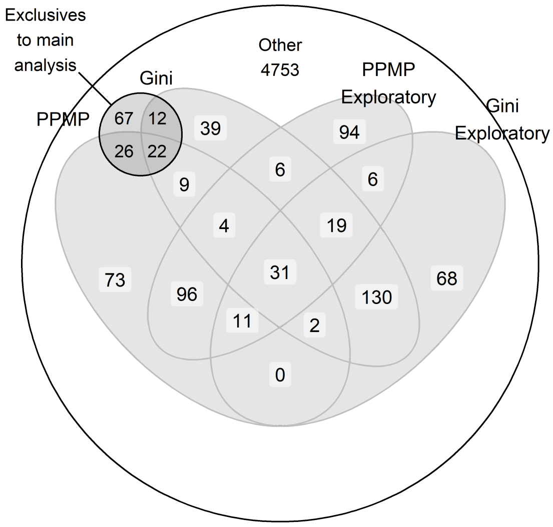

We have added description of the Venn diagram in the results section.

> Overlap between indices (PPMP *versus* Gini index) and analyses (principal *versus* sensitivity analyses) is presented in **Web-Appendix 7**. Among the 648 journals flagged as outliers either through the principal analyses, or through the sensitivity analyses, 299 (46.1%) are common to both analyses. Furthermore, qualitative analysis of the 100 randomly selected outlier journals identified through the sensitivity analyses is consistent with those for the principal analyses (see **Web-Appendix 6**).

We have added some comments in the discussion about this point (see the next points).

[27] "*The main characteristics of the 100 randomly selected outlier journals are presented*" A statistician should confirm random vs pseudo-random. Re: the discussion of journals "*reported in English.*" This is somewhat misleading since by nature the NLM is skewed towards English language journals.

Refer to answer **[19]**
Re: NLM. We have edited our discussion accordingly.

“On a random sample of outlier journals identified using the PPMP and/or the Gini index, we found that the prolific authors can be ‘established’ scientists with a relatively high h-Index (e.g. a median of 28), and that 60% of these most prolific authors were editors-in-chief or members of the journal’s editorial board. About half of these journals had a median journal impact factor of 2.9 (IQR 1.5 to 4.8). These journals generally presented a large self-citation ratio, meaning that some of them may have questionable practices by manipulating their impact factor. The other half of these journals did not have an impact factor, possibly indicating they were new journals joining the WoS. Even though WoS uses an extensive list of eligibility criteria, it is also possible that some of the new journals are predatory, journals that are known to have “leaked into” trusted sources [7]. Importantly, while NLM is skewed towards English language journals, several outlier journals were in other languages. These journals are likely to represent smaller communities with the possibility of closer interactions between researchers.”

[28] "*We identified errors in author identification for one journal, MMW-Fortschritte der Medizin (an outlier on the Gini index), where the most prolific 'author' was named 'Red', which seems to be a diminutive for 'Redaktion', possibly encompassing several physical individuals.*" Not sure how this would be considered "an error"? "Redaktion" appears to be German for "Editorial Staff." So these would more appropriately belong to the "unnamed" articles?

Exactly, these papers would rather be better fit in the ‘unnamed’ category as these were not related with an individual author. However, the meta-data contain such a generic name that does not correspond to a physical person. We see this as an ‘error’ as we initially only plan to consider real, physical unique human as an ‘author’. And indeed, when we explored the effect of the removal of ’Red’ as a valid author name, we effectively considered articles only authored by ‘Red’ as unnamed articles. We have slightly edited the text to make this clearer.

« Because there was sometimes more than one author with the same largest number of published articles, 108 ‘most prolific’ authors were identified in the 100 outlier journals. We identified errors in author identification for one journal, MMW-Fortschritte der Medizin (an outlier on the Gini index), where the most prolific ‘author’ was named ‘Red’, which seems to be a diminutive for ‘Redaktion’, possibly encompassing several physical individuals. When ‘Red’ was ruled out as a valid author name (i.e. these papers were considered has having no author), the next most prolific author was however a member of the editorial board, the PPMP increased very slightly from 7.5% of 4 920 authored articles to 7.6% of 4 553 authored articles, and the Gini index decreased very slightly from 0.637 to 0.616. »

[29] "*When 'Red' was ruled out as a valid author name, the next most prolific author was however a member of the editorial board,…*" For a journal with a highly active editorial board as demonstrated by the number of "Red" articles, would this be unusual or unseemly? Might it be possible that MMW-Fortschritte der Medizin has its own publishing emphasis?

It may not be surprising if 'Red' is only used when, for example, multiple editors produced the published element, and are still listed with their individual name when publishing a redaction production in solo. When excluding 'Red' as an author name, the most prolific author was a single physician. Beyond the case of this specific journal, this comment highlights the ambiguity that there is between:

- prolific editorial activity;
- and use of a research journal to boost one own publication record.

In our opinion, there is no good metric to differentiate both activities. As explained before, we have revised our manuscript to present more comprehensively the 2 analyses :

- On all articles (including editorials etc.) / main analysis;
- On 'research articles' and, here, we have refined our definition / sensitivity analysis.

In this new version, based on all the comments received, we have decided to add much more importance to the sensitivity analysis:

- We explored overlap with the main analysis;
- We extracted a new random sample for this analysis.

First, results of both analyses are similar with a slight reduction in the number of article in each journal (426, IQR 232 to 798 for sensitivity versus 500, IQR 262 to 964 for the main analysis) and only 127 journals out of 5468 which have to be excluded for having less than 50 authored articles between 2014 and 2019. The PPMP 95th percentile decreased slightly, from 10,6% to 8,8%, and the Gini index 95th percentile decreased marginly from 0,355 to 0,324.

Still, the overlap in identity of outliers is not perfect (see the Venn Diagram, ansewer **[26]**) suggesting that one should clearly make a distinction between prolific editorial activity and much more problematic behaviors. There is certainly a grey zone and we edited the discussion to make this much more explicit. We have added much more emphasis about this point in the discussion. See our response to the comments made about the discussion.

[30] How did the authors confirm that the same-named authors were indeed the same person? Did they cross-check affiliations, or just assume the same name in the same field would be the same person?

Refer to answer **[14]**

"Outliers were defined as journals with a PPMP value and/or the Gini index above their respective 95th percentiles in the principal analysis (i.e. on all articles). For pragmatic reasons a sample of 100 outlier journals was randomly selected (first sorted by full name, in alphabetic order, and randomly sampled using a random number generator with a seed arbitrarily set at 42; R function sample_n in dplyr package).<br>One reviewer (AS or FN or CL) manually extracted characteristics related to the journal impact factor (Web of Science: WoS), open access policies (WoS and Directory of Open Access Journals), open peer review policies (Publons - Clarivate), the most prolific authors' h-index (WoS) and presence and role (i.e. editor in chief or board member) on the editorial board of the journal (journal or publisher website). Where this information was available, we made a distinction between advisory boards (that were not considered in the analysis) and editorial boards. For the year 2019, the metrics 'self-citation boost' (i.e. number of self-citing articles over number of non-self-citing articles) and 'skewness & non-article inflation' (i.e. impact factor minus median of citations for an article, over median of citations for an article) was computed according to Ioannidis and Thombs [6]. Importantly, this extraction allowed for qualitatively exploring the possibility of homonyms between authors by comparing names, affiliations, research field, and all available qualitative information on NLM and WoS (using WoS Author search tool). When a doubt persisted, we used Google to identify the author at risk of homonymy and their credentials."

Discussion:

[31] "*In this comprehensive survey of 5 468 biomedical journals, we characterized several features of editor-author relationships among which were the following: (i) article output was sometimes dominated by the prolific contribution of one author or a group of authors, (ii) time lags to publication were in some instances shorter for these prolific authors and (iii) prolific authors were typically members of the journal's editorial board.*" Criteria for "*sometimes*" and "*typically*" are unclear. Additionally, as pointed out previously, the publication lag data was for less than half the journals considered in the sample, but was compared against figures for all the journals.

We agree, we, have edited our discussion. In this first part we describe our findings.

"In this comprehensive survey of 5 468 biomedical journals, we describe several features of editor-author relationships among which were the following: (i) an article output dominated by the prolific contribution of one author or a group of authors, (ii) with time lags to publication that were shorter for these prolific authors (when this information was available) and (iii) more than half of those prolific authors were members of the journal's editorial board."

In addition, please refer to answer **[25]**.

[32] "*We concluded that defining the top 5% nepotistic journals required the threshold to be set at up to 10.6% of articles published by the most prolific author.*" "for the purposes of this study" should be added. This study is insufficient for a general standard.

Thank you for mentioning that point, we made the following change in our effort to moderate our discussion.

In our study, the threshold to define the 95th percentile was 10.6% of articles published by the most prolific author. In absolute terms, we believe it is reasonable to closely inspect these journals as it may question the judgement of an editor where more than 10% of the published papers are authored by the same person.

[33] "*In absolute terms, we believe it is reasonable to question the judgement of an editor where more than 10% of the published papers are authored by the same person.*" This may be reasonable, but without at least a validated spot-check of some of these papers, this

statement appears to go well beyond what can be concluded from the findings. We note that a preprint of this manuscript has been the story of a news story in Science https://www.sciencemag.org/news/2021/02/journals-singled-out-favoritism that mentions the Didier Raoult oeuvre. Is there material there that could be cited?

Thanks for this comment. Please see our response to comment **[11]**. We now refer to the Science news story and we have edited this sentence.

"In our study, the threshold to define the 95th percentile was 10.6% of articles published by the most prolific author. In absolute terms, we believe it is reasonable to closely inspect these journals as it may question the judgement of an editor where more than 10% of the published papers are authored by the same person. To better characterize the lack of heterogeneity in authorship, we also computed the Gini index, for which the corresponding 95th percentile was 0.355 over five years and 0.20 when computed annually. This suggests that a broader time-frame allowed for more occasional authors to be recruited while maintaining the regular authors, revealing the latent heterogeneity."

[34] "*This suggests that a broader time-frame allowed for more occasional authors to be recruited while maintaining the regular authors, revealing the latent heterogeneity.*" Or revealed a beginning journal struggling to keep afloat long enough to attract a variety of authors and allow the "regular authors" to fade out to other publishing duties. Association is of course not causation.

Of course, we agree and this is really relevant. We edited the text. To note, there only 101 journals (1.9%) that begin its activity in 2015 or later, and 384 (7.0%) between 2010 and 2014.

"One possible explanation is that a broader time-frame allowed for more occasional authors to be recruited while maintaining the regular authors, revealing the latent heterogeneity. One other explanation is that some recent journals are relying on a group of authors on their first year before allowing these authors to move on to other publishing duties."

[35] "*If a journal publishes a very large number of papers, it becomes increasingly implausible that a single prolific author could account for 10% or more of them, as there is a natural upper limit to how many papers any one individual can author.*" While this is not an unreasonable conclusion to draw, it should be noted that senior researchers who oversee multiple projects may end up as authors on many papers, which naturally ups their publication count.

See our answer to point **[23]**.

"The PPMP and the Gini index explore complementary patterns of asymmetry in publishing patterns. The PPMP reflects author practices while the Gini is more sensitive to groups of highly prolific authors. The Gini index has one advantage over the PPMP, in that it is less constrained by the total number of articles published. If a journal publishes a very large number of papers (i.e. 1000 articles over 12 months), it becomes increasingly implausible that a single prolific author could account for 10% or more of them, as there is a natural upper limit to how many papers any one individual can author. Conversely, for a journal that publishes very few papers, an author could be identified as above the 95th centile with a relatively modest number of publications. In other words, there might be a masking effect in journals with higher rates of publishing causing lower publishing-rate journals to show a greater number of PPMPs. Further developments may consider breaking the journals into groups based on rates of publishing and/or using crude number of publications by the most prolific author."

Concerning this specific comment "While this is not an unreasonable conclusion to draw, it should be noted that senior researchers who oversee multiple projects may end up as authors on many papers, which naturally ups their publication count." We don't think that this is the better place to discuss it. We have edited another part of the discussion.

"We should beware of assuming that a hyper-prolific author is necessarily engaged in questionable publishing practices: some people are highly productive, and the speed with which good research can be completed is highly variable across research fields. Furthermore, authors may be represented in many papers because they play a key role in one aspect of the research, such as the statistical analysis, and senior researchers who oversee multiple projects may end up as authors on many papers. Similarly, shorter publication lags may occur simply because it is easier to find reviewers for eminent authors, or in a particular subject area, and/or because their expertise means that their papers require less revision."

[36] "*Our findings persisted when all articles were considered as well as when only 'journal articles' were considered (i.e. excluding articles explicitly referenced as editorial, correspondence or news articles), suggesting that editorials, correspondence and news, are not the only drivers of the indicators we explored.*" Did the authors confirm that the NLM labeling of type was accurate? PubMed relies on metadata from publishers, which has been shown to be incomplete or error-laden in a not insignificant number of cases.

As discussed in our response to point **[11]**, the labeling 'journal articles' was not sufficient and the risk of false positive results was too high. For instance, the Science news story picked up JADA (discussed earlier that was a false positive) but also to Journal of Enzyme Inhibition and Medicinal Chemistry that was is surely a problematic case as one may see by a qualitative exploration of the output of the most prolific author and chief editor.

Still, we believe that the risk of false positive is more problematic that the risk of false negative and we have therefore changed our definition of research articles (see point **[11]**). We now used an approach that was used in 2 previous papers.

Still, the risk of false positive exists and we have added more warning about this in the discussion section.

> "Our findings persisted when all articles were considered as well as when only 'research articles' were considered (i.e. excluding articles explicitly referenced as editorial, correspondence or news articles), suggesting that editorials, correspondence and news, are not the only drivers of the indicators we explored. Conversely, it is possible that the definition we used to identify research articles is not perfect and carries a risk of misclassification bias. However, analyzing both the overlap of PPMP and Gini and the overlap between both analysis populations cautions against making simple binary distinctions. When based on all articles, these metrics carry a risk of false positives, represented by active editors and/or professional writers. When based on research articles, it may miss problematic behaviors by certain editors. In other words, there is surely a grey zone; the proposed metrics are useful to delineate the big picture of medical journals behaviors, but the specific behaviors of these journals will necessarily deserve more fine-grained scrutiny in future. For instance, it will be interesting to see whether hyper-prolific authors publishing in their own journals adhere to COPE policy regarding conflicts of interest disclosures [8]. This was studied for authors of Cochrane reviews who are also editorial board members: the adherence to conflicts of interest policy was low [9]."

[37] "*We should beware of assuming that a hyper-prolific author is necessarily engaged in questionable publishing practices: some people are naturally highly productive, and the speed with which good research can be completed is highly variable across research fields.*" Is a "hyper-prolific author" different to a PPMP? Here, those authors are given a pass, but a few paragraphs prior these authors cast aspersions on the editors for allowing such prolific behavior.

In this paragraph, we don't want to give a "free pass" to any hyper-prolific behavior. Our intention was to add some nuance by explaining that in some occasions it may not be a problem. See our response to point **[33]**. We hope that our discussion is more coherent now.

"In our study, the threshold to define the 95th percentile was 10.6% of articles published by the most prolific author. In absolute terms, we believe it is reasonable to closely inspect these journals as it may question the judgement of an editor where more than 10% of the published papers are authored by the same person. There may be acceptable justification for such a pattern, but we cannot assume this. To better characterize the lack of heterogeneity in authorship, we also computed the Gini index, for which the corresponding 95th percentile was 0.355 over five years and 0.20 when computed annually. This suggests that a broader time-frame is most valuable for detecting lack of heterogeneity via the Gini index.

[38] "*It is also very important to complement the PPMP and the Gini index with the absolute numbers of papers authored by the most prolific authors, because some problematic journal behaviours could pass unnoticed when only these two indices are used.*" This suggests that the PPMP is not a "simple measure" as described elsewhere in the manuscript.

We agree and have changed this. When we used the term "simple", we were meaning that it was "simple" to compute and understand. However, we agree that this is not so simple to interpret this measure. Of course, no single metric can summarise a journal's quality, integrity, etc... At best, a variety of metrics may help but we recommend the same precautions that others have previously proposed about citation metrics: "*Different citation metrics may offer complementary insights, but one should carefully consider the assumptions that underlie their calculation*" [13]. We have edited the text accordingly.

See our response to point [4].

"We applied a preliminary approach to detect these 'self-promotion journals' in the field of infectious disease using a measure that is easy to compute: the proportion of contributions published in a journal by the most prolific author, i.e. the one who published the most articles in a given time period [1]."

And:

The two proposed indicators, and their current calculation, should therefore not be used indiscriminately but could rather serve as a screening tool for potentially problematic journals that may then require careful exploration of their editorial practices. In addition, by analogy with citation-based metrics [13], we believe no single metric can be sufficient but rather that different metrics can be complementary to inform about editorial behavior and that these metrics must not be used indiscriminately without considering all the identified limitations.

[13] Ioannidis JPA, Boyack K, Wouters PF. Citation Metrics: A Primer on How (Not) to Normalize. PLOS Biol. 2016;14: e1002542. doi:10.1371/journal.pbio.1002542

Limitations:

[39] "*but it may miss some finer points, especially concerning the quality of articles published in these journals.*" This is a significant limitation, as noted above, and deserves mention in the same breadth as other discussions of quality in the manuscript.

We have added some discussion about this point.

"Our descriptive and exploratory analyses, based on a large available database, provides information about the broad scene of 'nepotistic journals' but it may miss some finer points, especially concerning the quality of articles published in these journals. The quality of a scientific article is a difficult concept to measure and it cannot be easily summarized in quantitative metrics. We recommend a qualitative analysis of the papers published by the most prolific authors in journals flagged by these indicators, as well as an analysis of the relationship between this author and the editorial board."

[40] "*Some journals are registered without broad terms, requiring a manual pick-up by the NLM.*" This is unclear. If the authors are referring to Medline and article indexings - the Publisher applies to Medline. No BSTs are required for the application.

Refer to answer **[11]**.

[41] "*Similarly, because we restricted our search to journals publishing a minimum of 50 papers in the 2015-2019 period, we may have missed smaller journals with less professional editorial practices.*" Why is the assumption that smaller journals have less professional

editorial practices? If this is referring to editorial staff, which is not the same as "editorial practices," that should be made clear.

We have edited the text accordingly.

"Similarly, because we restricted our search to journals publishing a minimum of 50 papers in the 2015-2019 period, we may have missed smaller journals with less professional editorial staff and/or from publishers with different standards."

[42] "*Importantly, our automated calculations carry a risk of inaccuracy as a result of homonymy…. Our analysis of the random sample of outliers enabled a disambiguation procedure consisting in inspecting qualitatively the most prolific authors. Only 1 out of 108 "most prolific" authors within a given journal was considered to be at being at high risk of homonymy.*" What was the disambiguation procedure? If only looking at the ones already considered to be prolific, how does this method ensure that others may miss such a determination due to slight differences in the names used? (For example, Erin Nicole Potts-Kant has published as Erin Potts, Erin Kant, Erin N Potts-Kant, etc. Others have swapped first, middle and last names, with minor spelling changes, depending upon the journal.)

See our response to points [30]. We have added details in the method section. As disambiguation was explored for the most prolific author of a journal, homonyms risk identification were not carried back into analysis, as to retain comparability with other authors and don't perturb outliers randomisation.

"One reviewer (AS or FN or CL) manually extracted characteristics related to the journal impact factor (Web of Science: WoS), open access policies (WoS and Directory of Open Access Journals), open peer review policies (Publons - Clarivate), the most prolific authors' h-index (WoS) and presence and role (i.e. editor in chief or board member) on the editorial board of the journal (journal or publisher website). Where this information was available, we made a distinction between advisory boards (that were not considered in the analysis) and editorial boards. For the year 2019, the metrics 'self-citation boost' (i.e. number of self-citing articles over number of non-self-citing articles) and 'skewness & non-article inflation' (i.e. impact factor minus median of citations for an article, over median of citations for an article) was computed according to Ioannidis and Thombs [6]. Importantly, this extraction allowed for qualitatively exploring the possibility of homonyms between authors by comparing names, affiliations, research field, and all available qualitative information on NLM and WoS (using WoS Author

search tool). When a doubt persisted, we used Google to identify the author at risk of homonymy and their credentials."

The problem pointed in this comment is also about underestimation of PPMP. As answered in our previous response (point **[14]**), there is no perfect way to address this. We have edited the text accordingly.

"Importantly, our automated calculations rely on the articles identified through a NLM search and it is possible that not all journals list all articles within Pubmed. Although this is likely rare, we cannot exclude that certain authors use somewhat different name (e.g. change of name after marriage). We could not explore this bias that would have resulted in an underestimation of our indicators. More importantly, these calculations carry a risk of inaccuracy as a result of homonymy."

[43] "*Among these 108 authors, this procedure also enabled identification of the 6 most prolific authors, who were professional journalists for whom high productivity is of course not an indicator of any academic misconduct* [emphasis ours], as they are professionals paid by the journal and not academics. The two proposed indicators, and their current calculation, should therefore not be used indiscriminately but could rather serve as a screening tool for potentially problematic journals that may then require careful exploration of their editorial practices." This also suggests the PPMP is not a "simple measure," and casts doubt on the earlier statement that "In absolute terms, we believe it is reasonable to question the judgement of an editor where more than 10% of the published papers are authored by the same person."

See our responses to points **[11]**, **[33]** and **[38]**. We have revised our approach and we have added much more nuance.

[44] "*The proposed indices could add transparency in the editorial decision-making and peer review process of any journal*." We agree, provided the nuance and limitations are clearly reflected.

We hope that our previous answer and modifications are adequate. In our opinions, these were very helpful comment that helped to improve our manuscript. We hope that we have answered in a satisfying manner.

Reviewer #3:

The paper proposes and analyzes two interesting metrics for detecting possibly nepotistic practices in journals. Both, the PPMP and the Gini index, are easy to compute and to understand and they allow to identify outlier journals and authors than can be then double checked for editorial misconduct. The experimental design, data and code are available in OSF.

Thank you for this comments that summarize nicely the main message of our manuscript.

The analysis is rather descriptive and it would be great to see some statistical models in place (eg. when characterizing the profile of most prolific authors and of outlier journals).

Although I understand the pragmatic decision of restricting the analysis of outlier journals to 100, I think the work should better cover all 480 journals.

At this point, we don't have the resources to complete such analyses in a timely manner. We preferred to use our limited resources to extract and analyse a second sample of 100 outlier journals identified from the sensitivity analyses (see our answers to the other reviewers). Both samples were randomly selected. As anticipated by the academic editor, adding more observations would increase precision without modifying results. This is however something that we will consider for further work.

The authors should more explicitly describe the actual contribution of some co-authors of this study.

This was done:

C.L., F.N. and A.S. initiated the project and drafted the protocol. I.C, DM and DB critically revised the protocol. F.N. and A.S. extracted the data. A.S. analyzed the data. C.L., F.N. and A.S. wrote the first draft of the manuscript. I.C, DM and DB critically revised the manuscript. All authors approved final submitted version of the article.

COMMENTS FROM THE ACADEMIC EDITOR:

The report from the reviewers cover a lot of terrain in much detail. On top of this, there a couple of more general nature that I would like to give back as feedback that you might choose to use in a next version:

[1] Take reader by the hand with a bit more explanation at the somewhat more complex or less widely known concepts.

We kept this suggestion in mind when addressing comments of reviewer 2. For instance, we tried to provide more explanation in the introduction section, and to make sure that a broad audience may understand the concepts described. Please see our response to reviewer 2 point **[4]**.

[2] The figures are a good example - add perhaps 1-2 more sentences on what can be learned from the different figures, and how relate to each other.

This was done for all figures.

> "For the principal analyses based on all articles published during the 2015–2019 period, the PPMP median was 2.88% (IQR 1.71% to 4.91%) and the 95th percentile of the PPMP value was 10.6%. Figure 2A details the PPMP and numbers of published outputs for all journals. It also shows that the number of publications a prolific author in a given journal can result in different PPMP depending on the number of papers published in this journal. The most prolific author(s) in each journal published a median of 14 articles (IQR 8 to 25). For 1022 journals (19%), there was more than one author with the same largest number of published articles. Over the 2015–2019 period, the Gini index median was 0.183 (IQR 0.131 to 0.246), and the 95th percentile was 0.355. Figure 2B details the Gini indices and numbers of published outputs for all journals. Figure 2C presents the correlation between the PPMP and Gini indices. This correlation was 0.35 (95CI 0.33 to 0.37).
> For both indices, there were no meaningful differences between index values across years, with the 95th percentile ranging from 10.1% to 11.4% for the PPMP and 0.212 to 0.224 for the Gini index (Web-Appendix 2)."

And

"Figure 3 shows the scatter plot for all articles with the marginal density curve for the median of publication lag for the most prolific author(s) versus non-prolific authors, for each journal: for articles authored by the most prolific author(s), the distribution of the publication lag was skewed towards a shorter time-lag. Using a cut-off of 3 weeks for the median of publication lag, 277 (10.2%) of the journals had a median below this for articles by the most prolific author(s), 51 (1.9%) journals had a median below this for articles not by prolific author(s), and 38 (1.4%) journals had a median below this for both types of article (i.e authored by the most prolific author(s) or not). For the most prolific authors, publication lag decreased with the number of articles published as shown in Figure 4, not solely in outlier journals. The results of the sensitivity analyses based on 'journal articles' alone were consistent with those for all articles (Web-Appendix 5)."

Another example is that some concepts are used, yet only explained in detail in the methods. That is relatively late due to the lay out format of PLOS Biol. This means that some aspects of the methods have to be introduced with a couple of extra words in the intro/results or discussion section. (e.g. gini index, "journal articles")

Thank you for this advice. The manuscript has been reworked to facilitate understanding.

1/ Gini index: we have added more details in the introduction, especially about its use in bibliometry:

A highly prolific author who is an outlier on the PPMP measure in effect monopolizes a large proportion of a journal's publications. This analogy supports another, more complex measure, the Gini index [4], used in econometrics to describe resource distribution inequalities. This measure was recently applied in bibliometrics to explore inequality in authorship across authors publishing in high-impact academic medical journals [5]. Applied to our context, it could be used to quantify imbalances in the patterns of authorship within a journal: the values of the Gini index range from 0 (perfect equality in numbers of articles among authors) to 1 (major inequality).

Please note that this sentence was moved from the method section: "The values of the Gini index range from 0 (perfect equality in numbers of articles among authors) to 1 (major inequality)". It was therefore deleted from the methods section.

Complementary to the PPMP, the Gini index was used to explore inequality in the number of published articles related to more than one author. The Gini index for the number of publications by each author was calculated, with correction for the total number of authors (see formula and example in Web-Appendix 9)[3]. ~~The values of the Gini index range from 0 (perfect equality in numbers of articles among authors) to 1 (major inequality).~~

2/ Journal articles: we rather refer now to research articles which may be more easy to understand. In the introduction we make now the distinction between research articles and editorial, comments etc.). We hope that it is now more easy to follow.

"Research integrity matters across the research ecosystem. In this process, scientific journal editors are key actors that ensure the trustworthiness of the scientific publication process. But, paraphrasing Dr. Drummond Rennie's famous quote, who is guarding those guardians? [1] Some of our team (CL, IC, DM and FN) had doubts that anyone does such safe guarding in the case of New Microbes and New Infection (NMNI), an Elsevier journal, whose most prolific author, Didier Raoult, co-authored 32% of its 728 published papers [2]. NMNI's editor-in-chief and six additional associate editors of the journal work directly for, and report to, Raoult. Together, these editors authored 44% of the 728 papers published in the journal as of June 25, 2020. We suggested that such 'self-promotion journals' were "*a new type of illegitimate publishing entity, which could have certain key characteristics such as (i) a constantly high proportion of papers published by the same group of authors, (ii) relationships between the editors and these authors, and (iii) publication of low-quality research*" [2]. We applied a preliminary approach to detect these 'self-promotion journals' in the field of infectious disease using a measure easy to compute: the proportion of contributions published in a journal by the most prolific author, i.e. the one who published the most articles in a given time period [2]. In journals publishing more than 50 papers over 5 years, it was rare to see journals where a specific author published more than 10% of the papers, and indeed NMNI was a clear outlier. Note however, this is a crude measure as it is based on all published articles, whatever their type (research, letter, editorial, etc.) and therefore may give high scores for legitimate contributions by active editors.

Coincidently, one of our colleagues (DB) reported a similar analysis for the addiction subfield of psychology in a blog post [3]. This analysis focused on research articles only. She found a bimodal distribution of 'the percentage by the most prolific' measure, identical to that observed for NMNI, with only 3 out of 99 journals having a score over 8%. In two of these journals, the high score was attributable to the same individual, who was on the editorial board of the journal, and who had published together with the editor-in-chief. Bishop also noted that the

same method identified a journal editor, Johnny Matson, who had been found previously to be publishing copiously in journals edited by himself or other editors [3]. Furthermore, for many of these papers, indirect evidence of superficial or absent peer reviews was suspected because of the remarkably rapid turn-around, often within a week or less, between the dates recorded for submission and manuscript acceptance. This circumstantial evidence of unethical editorial practice can only be obtained, however, in journals that report these dates for published manuscripts."

[3] journal age is only touched upon lightly in the narrative - I would like to point out fro personal experience with several new journals that when a new journal starts, there is active solicitation of papers from EiC to EB members to have enough copy in the starting up phase - What can be said about this phenomenon in relation to these indices?

We agree and this is surely even more relevant concerning the Gini index. We have therefore added some words about this point in our discussion, in line with reviewer 2 comments (see reviewer 2 point **[34]**).

"One possible explanation is that a broader time-frame allowed for more occasional authors to be recruited while maintaining the regular authors, revealing the latent heterogeneity. One other explanation is that some recent journals are relying on a group of authors on their first year before allowing these authors to move on to other publishing duties."

[4] with regard to the suggestion of reviewer 3 to analyse a larger set of outlier journals I think it is important to note that while adding more observations will indeed increase precision, such a change will not affect the future decision of the PLOS Biol to publish or not publish.

As specified previously, a second sample of 100 outlier journals – identified from the sensitivity analyses – was randomly selected and was extracted. We thought that this would be much more important in order to address comments made by reviewer 2. We however don't have the resource to extract all outlier journals.

[5] The openness on the used method (registered analysis, explicit mention of deviations in planned analysis) is great to see. If you decide to add to or change the analysis, feel free to note that these changes came through peer review in order to keep your level of "open reporting on methods used" intact. NB the level of detail on the selection procedure for outlier selection is recommendable. The seed number chosen is as obvious as right.

Thank you for your positive response to our method.

Following the peer review, the protocol was indeed amended. We had to make some changes to our methods, based on the comments gathered during the peer review process. But, please note that all the changes were made and registered before any new data collection (changes since the precedent version of the protocol were highlighted and the amended protocol was registered before reanalysis (https://osf.io/6evmz/). We have also edited the manuscript accordingly to make all these changes clear and transparent. We must that adopting such a process was quite long and we apologize in the delay for answering. However, in our opinion, this is some example that a thorough peer review process can hardly be done in less than 3 weeks. We sincerely thank you for your patience and for your interest in this manuscript.
