# Supplementary material for: A survey of biomedical journals to detect editorial bias and nepotistic behavior
Source: PLoS Biol. 2021 Nov 23;19(11):e3001133. doi: 10.1371/journal.pbio.3001133 (PMC8610247; doi:10.1371/journal.pbio.3001133)
Supplement: Attachment — Submitted filename: renamed_0f319.pdf [file pbio.3001133.s014.pdf]

Dear Dr. Roberts,

Thank you for your consideration of our work and for inviting us to submit this revised manuscript. Our point-by-point responses to the editorial and reviewer comments are described below. Changes to the initial version of the manuscript are highlighted in the revised version.

We believe that our manuscript has been considerably improved as a result of these two revisions, and hope that our revised manuscript "***A survey of biomedical journals to detect editorial bias and nepotistic behavior***" is acceptable for publication in PLOS Biology.

Yours sincerely,

Alexandre Scanff, Florian Naudet, Ioana Cristea, David Moher, Dorothy VM Bishop, Clara Locher

---

COMMENTS FROM THE EDITOR:

Based on the reviews, we will probably accept this manuscript for publication, provided you satisfactorily address the remaining minor point raised by reviewer #4 and the following:
Thank you for your interest in our manuscript.

[1] Please could you choose a more explicit and appealing title? We suggest the following ideas (assuming these are supported by your findings): "Editorial bias and nepotistic behaviour detected in 10% of biomedical journals" or "Nepotistic behaviour detected in 10% of biomedical journals" or "A survey of biomedical journals to detect editorial bias and nepotistic behavior." I should also say that we like to avoid punctuation in titles.
Thank you for you sugestions. The tittle has been modified: A survey of biomedical journals to detect editorial bias and nepotistic behavior.

[2] Please could you supply a blurb, as instructed in the submission form?

We suggest the following sentence: To highlight questionable editorial behaviors, this study explores the relationship between hyperprolific authors and a journal's editorial team.

[3] Please attend to the request from reviewer #4. Keep the Materials and Methods section in its current place, but address the reviewer's concern by adding a description of the Gini index earlier in the manuscript in order to help the reader appreciate the implications of its use more fully.

An initial description of the Gini index is given in the introduction section: "Applied to our context, it could be used to quantify inequalities in the patterns of authorship within a journal: the values of the Gini index range from 0 (perfect equality in numbers of articles among authors) to 1 (major inequality)."

To facilitate understanding, we also added a sentence in the Results section, just before presenting the results on Gini index. We also refer to a supporting figure –**S2 Fig. Formula and example to explain the Gini index**– that explains with an easy example how to calculate and interpret the Gini index.

In order not to burden the material and methods section, we prefer to keep detail explanation of the Gini index in the Supporting Figure 2. However, we have extended the caption of this Figure in order to provide all the essential information on Gini index calculation and interpretation.

Results section

For the principal analyses based on all articles published during the 2015–2019 period, the PPMP median was 2.88% (IQR 1.71% to 4.91%) and the 95th percentile of the PPMP value was 10.6%. Figure 2A details the PPMP and numbers of published outputs for all journals. It also shows that the number of publications by a prolific author in a given journal can result in different PPMP depending on the number of papers published in this journal. The most prolific author(s) in each journal published a median of 14 articles (IQR 8 to 25). For 1 022 journals (19%), there was more than one author with the same largest number of published articles. To assess authorship disparity from not only a single highly prolific author but from a group of authors, we also computed the Gini index, a measure of the degree of unequal distribution of authorship within a journal (**S2 Fig**). Gini index range from 0 to 1, with smaller values indicating a more equal distribution of articles across authors and higher values representing greater inequality. Over the 2015–2019 period, the Gini index median was 0.183 (IQR 0.131 to 0.246), and the 95th percentile was 0.355. Figure 2B details the Gini indices and numbers of published outputs for all journals. Figure 2C presents the correlation between the PPMP and Gini indices. This correlation was 0.35 (95CI 0.33 to 0.37).

S2 Fig caption.

Formula and example to understand the Gini index. We investigated the level of inequality in the distribution of authorship among authors using the Gini index. This statistical measure

is derived from the Lorenz curve and is widely used in econometrics to describe income or wealth inequalities in a given population. In our study, 'income' corresponds to the number of articles signed by authors in a given journal and the 'population' is all authors who have published at least one article between 2015 and 2019 in the journal. The Lorenz curve is a graphical representation of the ranked distribution of the cumulative percentage of authors on the abscissa versus the cumulative percentage of authorship distributed along the ordinate axis. In case of complete equality across author (i.e. each author within a journal has published the exact same number of articles), the Lorenz curve would follow the 45 degree diagonal. The further inequality increases (i.e. one author or a group of authors published more articles than others authors), the further the Lorenz curve moves away from this diagonal of equal distribution.

The Gini index is a measure of the area between the diagonal of equal distribution and the Lorenz curve, corrected for the number of authors $\frac{n}{n-1}$. In practice, the calculation formula is $[\frac{2\sum i y_i}{n\sum y_i} - \frac{n+1}{n}]\frac{n}{n-1}$ where n is the total number of authors, and y_i is the number of articles published by author i, with authors sorted in non-decreasing order of article numbers. The Gini index ranges from 0 to 1, with smaller values indicating a more equal distribution of articles across authors and higher values representing greater inequality. As expressed in its formula, the Gini index calculation gives a higher weight to extreme positive values, and may be comparable in interpretation to a normalised root-mean-square error against an expected distribution of 'all authors appear exactly the same number of times'.

As a toy example, hypothetical cases of distribution of authorship between 3 authors totalling 24 authorships (left), with corresponding Lorenz curve and Gini index (right) are shown in the figure below. The dark blue line represents the diagonal of equal distribution (scenario 1) and other lines represents Lorenz curve of different scenarios of inequality (scenarios 2–5). The Gini formula counts the times an author's name occurs, but does not distinguish between papers contributed by different authors. Thus, the scenario 1 shown here could occur with 8 articles published in common by the 3 authors, or 4 articles published in common and each of the 3 separately publishing 4 other articles.

[4] We note that your financial statement currently says "The author(s) received no specific funding for this work" - please can you confirm that this is correct?

We confirm that we did not received funding for this work.

[5] We note that your supplementary Figures currently have rather confusing names. Please re-number them Figs S1, S2, S3, etc., provide legends for them, and cite them in the text.

File have been renamed as suggested and in the text. Web-Appendix 1 has been modified by **S1 Fig**, Web-Appendix 2 by **S1 Table**, Web-Appendix 3 by **S2 Fig**, etc. They are all cited in the text.
Furthermore, captions for figures were moved under the paragraph where the figure is first cited, figure titles were added and captions for Supporting Information were added at the end of the manuscript file.

Fig 1. **Flowchart of included journals.** Selection flowchart for journals labelled with at least one 'Broad Subject Term' in the National Library of Medicine (NLM).
Fig 2. **PPMP and Gini index among all articles**. Distribution of Percentage of Papers by the Most Prolific (PPMP) author(s) (A) and Gini index (B) in relation to journal size, and comparison between the PPMP and the Gini index (C), across all articles published by all journals in the United States National Library of Medicine catalog having at least one Broad Subject Term and having published at least 50 authored articles between 2015 and 2019. The data underlying this Figure may be found in https://osf.io/6e3uf/.
Fig 3. **Publication lag**. Distribution of the publication lag median for the subgroup of 2 725 (49.8%) journals reporting submission and publication dates. Publication lag median (in days) are presented for articles signed by the most prolific authors compared to the articles without any of the most prolific authors (with marginal density plot of distributions). The data underlying this Figure may be found in https://osf.io/6e3uf/.
Fig 4. **Publication lag for articles with at least one most prolific author.** Distribution of publication lag (in days) and number of articles authored for each of the most prolific authors, for all article types (with marginal density plot of distributions) for the subgroup of 2 725 (49.8%) journals reporting submission and publication dates. The data underlying this Figure may be found in https://osf.io/6e3uf/.

[6] We believe that the OSF deposition contains data and scripts sufficient to re-create all of the main and supplementary Figures. Can you confirm that this is the case? Please cite the location of the data clearly in each relevant main and supplementary Figure legend, e.g. "The data underlying this Figure may be found in https://osf.io/6e3uf/"
We confirmed that data and code deposited on OSF enable are sufficient to re-create all of the figures. The following sentence has been added to Figure legend: The data underlying this Figure may be found in https://osf.io/6e3uf/.

[7] Please re-write the Abstract in the more verbose format that is standard for our journal. I also notice that something has gone wrong with the second sentence of the Abstract.
The abstract has been modified.

Background: Alongside the growing concerns regarding predatory journal growth, other questionable editorial practices have gained visibility recently. Among them, we explored the usefulness of the Percentage of Papers by the Most Prolific author (PPMP) and the Gini index (level of inequality in the distribution of authorship among authors) as tools to identify journals that may show favoritism in accepting articles by specific authors.
Methods and findings: We examined whether the PPMP, complemented by the Gini index, could be useful for identifying cases of potential editorial bias, using all articles in a sample of 5 468 biomedical journals indexed in the National Library of Medicine. For articles published between 2015 and 2019, the median PPMP was 2.9%, and 5% of journal exhibited a PPMP of 10.6% or more. Among the journals with the highest PPMP or Gini index values, where a few authors were responsible for a disproportionate number of publications, a random sample was manually examined, revealing that the most prolific author was part of the editorial board in 60 cases (61%). The papers by the most prolific authors were more likely to be accepted for publication within 3 weeks of their submission. Results of analysis on a subset of articles, excluding non-research articles, were consistent with those of the principal analysis.
Conclusions In most journals, publications are distributed across a large number of authors. Our results reveal a subset of journals where a few authors, often members of the editorial board, were responsible for a disproportionate number of publications. To enhance trust in their practices, journals need to be transparent about their editorial and peer review practices.

REVIEWERS' COMMENTS:

Reviewer #2:

[identify themselves as Ivan Oransky and Alison Abritis]

We appreciate how seriously the authors have taken the suggestions of all of the reviewers, which indicates a high level of integrity in the work. The paper is much improved, and we are happy to recommend acceptance.

We would still recommend that the editors have a statistical expert review the methods, but we leave that to their discretion.

We sincerely appreciate all of your valuable comments and suggestions which have strongly improved our paper.

Reviewer #4:

Let me start by stating that in scientometrics/bibliometrics, the Gini-index is used more frequently, as a measure of concentration. I have personally used this as a measure of concentration with respect to field orientation, in other words, to what extent is a unit more mono-or interdisciplinary, and confront and correlate that with MRC research grant peer review assessments.

With respect to this paper, a number of things strike me, in the first place the order of the sections in the manuscript. I would expect the Methods section before the Results section, which would mean that one would read about the used measures, among which the Gini-index, before reading results. This is unfortunate, as one now has to except or assume the meaning of the Gini-index, and how it work, and what it does. In the Methods section I think the presentation of the Gini-index is not sufficient, reading the section I did not get a clear view on how this index works, how it is calculated, how it is applied in the dataset that is collected, and also not how this affects the outcomes. I think the authors should add such a paragraph to the manuscript, as many readers of the paper will probably be not very familiar with the index, so a further explanation helps better understand the outcomes of the study. One would then also create a better understanding of how the Gini-index relates to the PPMP-indicator presented in the study, as well as the way these two measures are correlated, and how to interpret their correlation.

I liked the study and the paper, as it deals with an important research integrity issue, namely that of authorship, and everything that can go wrong there, which often remains invisible among all questionable research practices.

Thank you for your interest in our manuscript and for your suggestions.
Concerning the order of the sections in the manuscript, we follow PLOS Biology instructions.
An initial description of the Gini index is given in the introduction section: "Applied to our context, it could be used to quantify inequalities in the patterns of authorship within a journal: the values of the Gini index range from 0 (perfect equality in numbers of articles among authors) to 1 (major inequality)."
To facilitate understanding, we also added a sentence in the Results section, just before presenting the results on Gini index. We also refer to a supporting figure **–S2 Fig. Formula and example to explain the Gini index–** that explains with an easy example how to calculate and interpret the Gini index.
In order not to burden the material and methods section, we prefer to keep detail explanation of the Gini index in the Supporting Figure 2. However, we have extended the caption of this

Figure in order to provide all the essential information on Gini index calculation and interpretation.

Results section

For the principal analyses based on all articles published during the 2015–2019 period, the PPMP median was 2.88% (IQR 1.71% to 4.91%) and the 95th percentile of the PPMP value was 10.6%. Figure 2A details the PPMP and numbers of published outputs for all journals. It also shows that the number of publications by a prolific author in a given journal can result in different PPMP depending on the number of papers published in this journal. The most prolific author(s) in each journal published a median of 14 articles (IQR 8 to 25). For 1 022 journals (19%), there was more than one author with the same largest number of published articles. To assess authorship disparity from not only a single highly prolific author but from a group of authors, we also computed the Gini index, a measure of the degree of unequal distribution of authorship within a journal (**S2 Fig**). Gini index range from 0 to 1, with smaller values indicating a more equal distribution of articles across authors and higher values representing greater inequality. Over the 2015–2019 period, the Gini index median was 0.183 (IQR 0.131 to 0.246), and the 95th percentile was 0.355. Figure 2B details the Gini indices and numbers of published outputs for all journals. Figure 2C presents the correlation between the PPMP and Gini indices. This correlation was 0.35 (95CI 0.33 to 0.37).

S2 Fig caption.

Formula and example to understand the Gini index. We investigated the level of inequality in the distribution of authorship among authors using the Gini index. This statistical measure is derived from the Lorenz curve and is widely used in econometrics to describe income or wealth inequalities in a given population. In our study, ‘income’ corresponds to the number of articles signed by authors in a given journal and the ‘population’ is all authors who have published at least one article between 2015 and 2019 in the journal. The Lorenz curve is a graphical representation of the ranked distribution of the cumulative percentage of authors on the abscissa versus the cumulative percentage of authorship distributed along the ordinate axis. In case of complete equality across author (i.e. each author within a journal has published the exact same number of articles), the Lorenz curve would follow the 45 degree diagonal. The further inequality increases (i.e. one author or a group of authors published more articles than others authors), the further the Lorenz curve moves away from this diagonal of equal distribution.

The Gini index is a measure of the area between the diagonal of equal distribution and the Lorenz curve, corrected for the number of authors $\frac{n}{n-1}$. In practice, the calculation formula is

$[\frac{2\sum iy_i}{n\sum y_i} - \frac{n+1}{n}]\frac{n}{n-1}$ where n is the total number of authors, and y_i is the number of articles published by author i, with authors sorted in non-decreasing order of article numbers. The Gini index ranges from 0 to 1, with smaller values indicating a more equal distribution of articles across authors and higher values representing greater inequality. As expressed in its formula, the Gini index calculation gives a higher weight to extreme positive values, and may be comparable in interpretation to a normalised root-mean-square error against an expected distribution of 'all authors appear exactly the same number of times'.

As a toy example, hypothetical cases of distribution of authorship between 3 authors totalling 24 authorships (left), with corresponding Lorenz curve and Gini index (right) are shown in the figure below. The dark blue line represents the diagonal of equal distribution (scenario 1) and other lines represents Lorenz curve of different scenarios of inequality (scenarios 2–5). The Gini formula counts the times an author's name occurs, but does not distinguish between papers contributed by different authors. Thus, the scenario 1 shown here could occur with 8 articles published in common by the 3 authors, or 4 articles published in common and each of the 3 separately publishing 4 other articles.
